# Overexpression of a methyl-CpG-binding protein gene *OsMBD707* leads to larger tiller angles and reduced photoperiod sensitivity in rice

**DOI:** 10.1186/s12870-021-02880-3

**Published:** 2021-02-18

**Authors:** Mengyu Qu, Zhujian Zhang, Tingmin Liang, Peipei Niu, Mingji Wu, Wenchao Chi, Zi-Qiang Chen, Zai-Jie Chen, Shubiao Zhang, Songbiao Chen

**Affiliations:** 1 Marine and Agricultural Biotechnology Laboratory, Institute of Oceanography, Minjiang University, Fuzhou, 350108 China; 2 Biotechnology Research Institute, Fujian Academy of Agricultural Sciences, Fuzhou, 350003 China; 3 College of Agriculture, Fujian Agriculture and Forestry University, Fuzhou, 350002 China

**Keywords:** Rice, MBD, Tiller angle, Flowering time, Photoperiod sensitivity, Overexpression

## Abstract

**Background:**

Methyl-CpG-binding domain (MBD) proteins play important roles in epigenetic gene regulation, and have diverse molecular, cellular, and biological functions in plants. MBD proteins have been functionally characterized in various plant species, including *Arabidopsis*, wheat, maize, and tomato. In rice, 17 sequences were bioinformatically predicted as putative MBD proteins. However, very little is known regarding the function of MBD proteins in rice.

**Results:**

We explored the expression patterns of the rice *OsMBD* family genes and identified 13 *OsMBDs* with active expression in various rice tissues. We further characterized the function of a rice class I MBD protein OsMBD707, and demonstrated that OsMBD707 is constitutively expressed and localized in the nucleus. Transgenic rice overexpressing *OsMBD707* displayed larger tiller angles and reduced photoperiod sensitivity—delayed flowering under short day (SD) and early flowering under long day (LD). RNA-seq analysis revealed that overexpression of *OsMBD707* led to reduced photoperiod sensitivity in rice and to expression changes in flowering regulator genes in the *Ehd1-Hd3a*/*RFT1* pathway.

**Conclusion:**

The results of this study suggested that OsMBD707 plays important roles in rice growth and development, and should lead to further studies on the functions of OsMBD proteins in growth, development, or other molecular, cellular, and biological processes in rice.

**Supplementary Information:**

The online version contains supplementary material available at 10.1186/s12870-021-02880-3.

## Background

DNA methylation, a conserved epigenetic modification in plant and animal genomes, plays an important role in genome stability, genomic imprinting, and gene expression regulation, and exerts effects on various aspects of plant and animal cellular and developmental processes [1, 2]. Methyl-CpG-binding domain (MBD) proteins emerge as important interpreters of DNA methylation marks [3]. MBD proteins can bind to methylated DNA and recruit chromatin remodeling factors, histone deacetylases and methylases to regulate gene expression [4–6]. Numerous studies in mammals revealed that mutations or abnormal expression of MBD proteins occur in many neurological diseases and cancers [7]. For example, mutations in the human methyl-CpG-binding protein 2 (MeCP2) cause a postnatal neurodevelopmental disorder Rett syndrome [8], demonstrating that MBD proteins play important roles in maintaining a normal epigenetic and cellular homeostasis.

Plant MBD proteins have been identified based on amino acid sequence similarity with mammalian MBDs [9, 10]. The *Arabidopsis* genome encodes 13 MBD domain-containing proteins [11]. Studies of the *Arabidopsis* MBDs showed that plant MBDs can bind to methylated or unmethylated DNA [10, 12], and interact with chromatin-modifying or transcriptional complexes, such as the histone deacetylase AtHDA6 [10], the histone methyltransferase AtPRMT11 [13], the histone acetyltransferase IDM1 and the alpha-crystallin domain protein ROS5/IDM2 in the DNA demethylation pathway [14–16], the RNA binding proteins AtRPS2C, AtAGO4 and AtNTF2 which are involved in the RNA-mediated gene silencing pathway [17], or the chromatin remodelers CHR11/CHR17 and chromatin remodeling complex SWR1 subunit PIE1 and ARP6 which are involved in histone H2A.Z deposition [18, 19]. The *Arabidopsis* MBDs play important roles in diverse molecular and biological processes. For example, knockdown of *AtMBD6* or *AtMBD10* leads to disturbed nucleolar dominance in *Arabidopsis suecica* [20]; mutation in *AtMBD8* causes late flowering in the C24 ecotype [21]; mutation in *AtMBD9* promotes early flowering and enhances shoot-branching in *Arabidopsis* [22, 23]; and knockdown of *AtMBD11* results in *Arabidopsis *plants with morphological and developmental abnormalities [9].

In addition to the studies in the model plant *Arabidopsis*, several recent researches have shown the important roles of MBD proteins in different plant species. The wheat *TaMBD2 *homoeologous genes were significantly induced by salt stress, suggesting their potential functions in stress responses [24]. *TaMBD6 *was highly responsive to prolonged chilling, suggesting that TaMBD6 was potentially involved in regulating the developmental transition from vegetative to reproductive stages in wheat [25]. The maize ZmMBD101 was identified to be required for maintaining Mutator (Mu) elements chromatin in maize [26]. In tomato, SlMBD5 was identified to interact with the CUL4-DDB1-DET1 complex, which was involved in the transcriptional activation of downstream genes [27]. Overexpressing of the *SlMBD5* gene leads to dark fruit color and dwarf phenotype in transgenic plants [27]. A number of *MBD* genes in four different solanaceae species have been observed to be differentially induced or suppressed during fruit development or abiotic stress responses, suggesting their roles involved in these processes [28].

Rice (*Oryza sativa* L.) is one of the most important crops worldwide. In the rice genome, 17 genes were bioinformatically predicted to encode putative MBD proteins and were designated as *OsMBD701* to *OsMBD718* [11]. However, very little is known regarding the functions of OsMBD proteins in rice. In this study, we explored the expression patterns of the predicted *OsMBD* family genes and detected 13 *OsMBDs* with active expression in various rice tissues. We further performed functional study of a rice class I MBD protein, OsMBD707, and revealed that overexpression of *OsMBD707* resulted in larger tiller angles and reduced photoperiod sensitivity in rice. Our results suggested that OsMBD707 plays biologicalroles in rice growth and development.

## Results

### Differential expression patterns of 13 *OsMBD *family genes in rice tissues

A bioinformatics analysis has identified 17 putative OsMBD proteins in rice [11]. Considering the annotation updates of the rice genome over the past decades, in this study, we verified the predicted *OsMBD *family genes by searching the National Center for Biotechnology Information (NCBI) and MSU Rice Genome Annotation Project (RGAP) databases. Our search revealed 15 genes in both NCBI and RGAP databases matching with previously predicted *OsMBD701*, *OsMBD703*, *OsMBD704*, *OsMBD705*, *OsMBD706*, *OsMBD707*, *OsMBD708*, *OsMBD709*, *OsMBD710*, *OsMBD711*, *OsMBD713*, *OsMBD714*, *OsMBD715*, *OsMBD717*, and *OsMBD718*, respectively (Additional file 1: Table S1). However, no NCBI RAP locus or MSU RGAP locus has been identified that match with *OsMBD712* or *OsMBD716* (Additional file 1: Table S1). In addition, a RAP locus *Os04g0192775* and a MSU RGAP locus *LOC_Os04g11510* were retrieved as genes putatively encoding MBD-containing proteins (Additional file 1: Table S1).

**Fig. 1:**
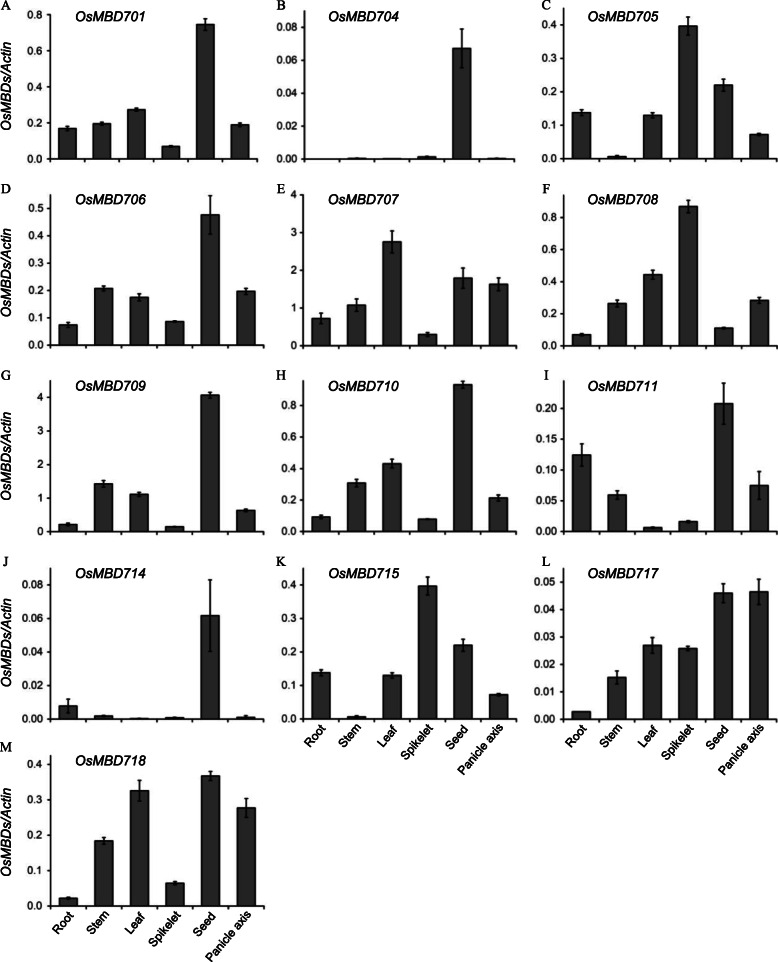
Expression profiles of the *OsMBD* family genes in various tissues of rice. **a**. *OsMBD701*. **b**. *OsMBD704*. **c**. *OsMBD705*. **d**. *OsMBD706*. **e**. *OsMBD707*. **f**. *OsMBD708*. **g**. *OsMBD709*. **h**. *OsMBD710*. **i**. *OsMBD711*. **j**. *OsMBD714*. **k**. *OsMBD715*. **l**. *OsMBD717*. **m**. *OsMBD718*. Relative expression levels of *OsMBDs* in root, stem, leaf, spikelet, seed, and panicle axis were determined by qRT-PCR. The rice *Actin* gene was used as an internal control

To understand the expression patterns of the *OsMBD* family genes, we first searched the digital expression values of *OsMBDs* in the RGAP database (http://rice.plantbiology.msu.edu/). Among the 17 putative OsMBD-encoding genes, *OsMBD701*, *OsMBD704*, *OsMBD705*, *OsMBD706*, *OsMBD707*, *OsMBD708*, *OsMBD709*, *OsMBD710*, *OsMBD711*, *OsMBD715*, *OsMBD717*, and *OsMBD718* showed differential expression in various tissues. However, there was no detectable expression value of *Os04g0192775* and *LOC_Os04g11510* in all 13 different tissues, and the predicted *OsMBD703*, *OsMBD713*, and *MBD714* showed very low FPKM (expected Fragments Per Kilobase of transcript per Million fragments sequenced) values only in callus, seed-5 days after pollination (DAP), or anther, respectively, but no detectable FPKM value in other tissues (Additional file 2: Table S2). We further performed quantitative RT-PCR (qRT-PCR) to validate the expression patterns of the *OsMBD* family genes in the roots, stems, leaves, spikelets, seeds, and panicle axes of rice plants. qRT-PCR results were nearly consistent with the digital expression database. Among the 17 putative *OsMBDs*, no transcripts of the predicted *OsMBD703*, *OsMBD713*, *Os04g0192775*, or *LOC_Os04g11510* were detected in tested tissues (Additional file 1: Table S1). While *OsMBD704* and *OsMBD714* were detected to be preferentially expressed in the seeds, *OsMBD701*, *OsMBD705*, *OsMBD706*, *OsMBD707*, *OsMBD708*, *OsMBD709*, *OsMBD710*, *OsMBD711*, *OsMBD715*, *OsMBD717*, and *OsMBD718* were observed to be differentially expressed in various tissues (Fig. 1).

### OsMBD707 is constitutively expressed and localized in the nucleus

**Fig. 2:**
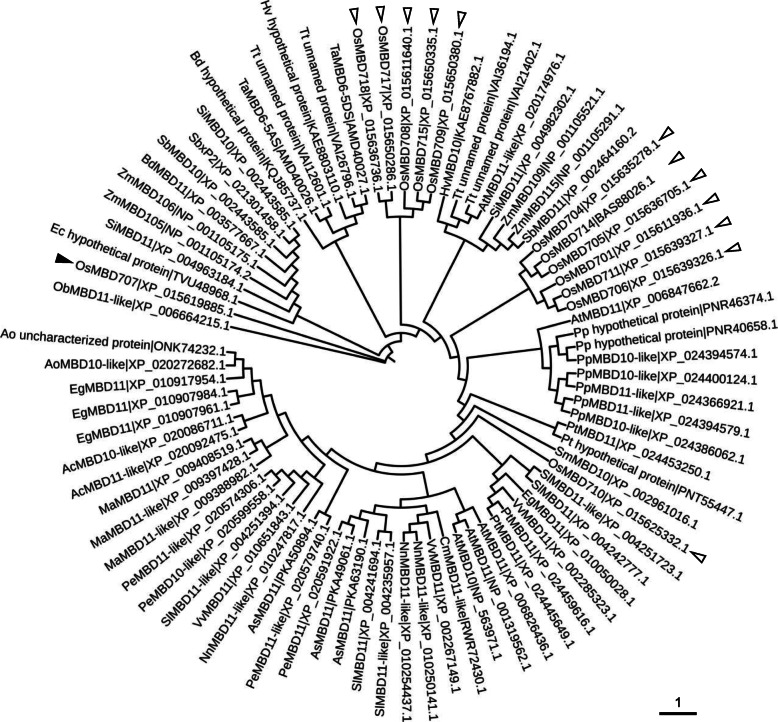
Phylogenetic analysis of OsMBD707. Phylogenetic tree was constructed using the neighbor-joining algorithm with 1000 bootstrap replicates. OsMBD707 is indicated by a black triangle. Other OsMBDs are indicated by empty triangles. Only the 13 OsMBDs with active expression detected by qRT-PCR were included in the phylogenetic analysis

**Fig. 3:**
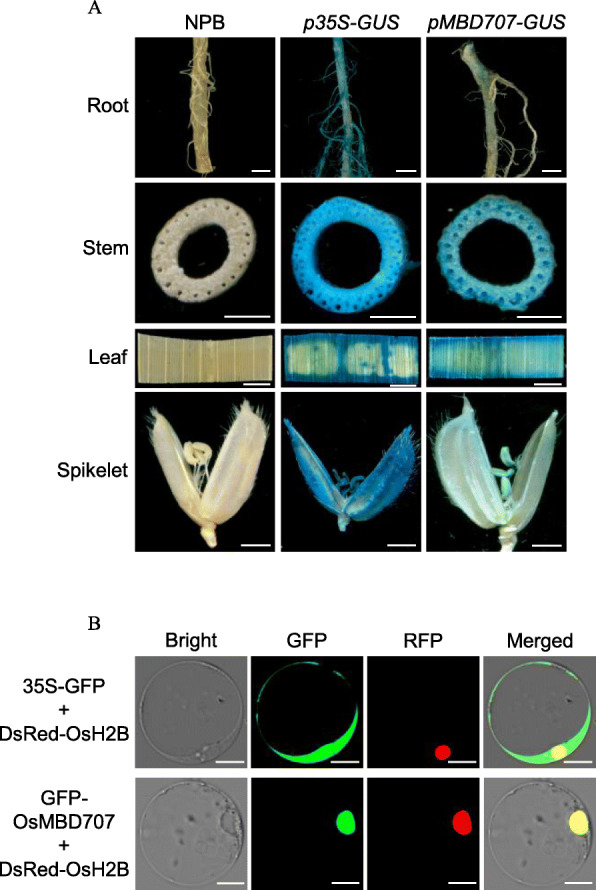
Expression pattern of *OsMBD707* and subcellular localization of OsMBD707. **a**. Histochemical GUS staining of the roots, stems, leaves, and spikelets of rice plant transformed with the *OsMBD707* promoter-*GUS* fusion construct. NPB, nontransgenic wild-type Nipponbare; *p35S-GUS*, rice plant transformed with a *CaMV 35S* promoter-*GUS* construct; *pMBD707*-*GUS*, riceplant transformed with the *OsMBD707 *promoter-GUS construct. Scale bar, 2 mm. **b**. Subcellular localization of the GFP-OsMBD707 fusion in rice protoplasts. 35S-GFP + DsRed-OsH2B, rice protoplast co-transfected with a *GFP* alone construct and a nucleus marker construct *DsRed-OsH2B*; GFP-OsMBD707 + DsRed-OsH2B, rice protoplast co-transfected with the *GFP-OsMBD707* fusion construct and *DsRed-OsH2B*; Bright, imagestaken under bright field; GFP, images taken under green fluorescence; RFP, images taken under red fluorescence; Merged, images merged from bright filed and green fluorescence channels. Scale bar, 10 μm

Rice OsMBDs could be divided into six classes [11]. In the present study, we focused on functional analysis of OsMBD707, which belongs to class I and is the only member of class I [11]. Phylogenetic analysis of OsMBD707 and closely related MBDs in various plant species showed that OsMBD707 was clustered together with ObMBD11-like in *Oryza brachyantha*, hypothetical protein TVU48968.1 in *Eragrostis curvula*, SiMBD10 and SiMBD11 in *Setaria italica*, ZmMBD105 and ZmMBD106 in *Zea mays*, BdMBD11 in *Brachypodium distachyon*, and SbMBD10 and SbxP2 in *Sorghum bicolor* (Fig. 2). qRT-PCR analysis showed that the mRNA of *OsMBD707* was expressed in various tissues, although the transcript level in the spikelets was relatively low (Fig. 1). A 1931-bp promoter fragment upstream of the translational start of *OsMBD707* was cloned and fused with the *GUS* reporter gene. Histochemical staining of rice plants transformed with the *OsMBD707* promoter-*GUS* fusion construct showed that GUS was expressed throughout the tested tissues, including the roots, stems, leaves, and spikelets, although the expression level was weaker than that of *35S* promoter-*GUS* transgenic plants (Fig. 3a). Overall, these results indicated that *OsMBD707* is constitutively expressed in various rice tissues.

*OsMBD707* was predicted to generate two alternative transcripts, *XM_015764399.1/LOC_Os12g42550.1*, and *XM_015764400.2*/*LOC_Os12g42550.2* (Additional file 3: Figure S1A, B). We performed RT-PCR to clone the cDNA fragment of *OsMBD707*, but obtained only *XM_015764399.1/LOC_Os12g42550.1*. An RT-PCR using primers (Additional file 3: Figure S1A) designed to distinguish the two predicted alternative transcripts was further performed. Consistently, only *XM_015764399.1/LOC_Os12g42550.1* was detected in all tested tissues, including the roots, stems, leaves, spikelets, seeds, and panicle axes (Additional file 3: Figure S1C, D), suggesting that there is only one isoform, LOC_Os12g42550.1, of OsMBD707. To explore the subcellular localization of OsMBD707, we performed transient expression of a *GFP-OsMBD707* fusion construct in rice protoplasts. Microscopy revealed that the fluorescence signal of GFP-OsMBD707 was inside the nucleus region, which was merged with the signal of the nucleus DsRed-OsH2B marker [29] (Fig. 3b). This result demonstrated that OsMBD707 is a nuclear-localized protein which is consistent with a function as a methyl-CpG-binding protein.

### Overexpression of *OsMBD707* causes larger tiller angles and reduced photoperiod sensitivity in rice

**Fig. 4:**
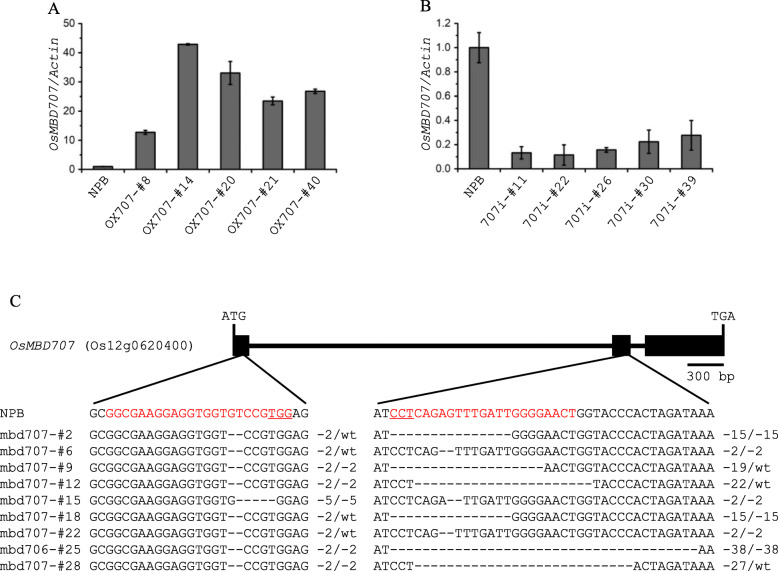
Molecular characterization of *OsMBD707*-overexpression, −knockdown and -frameshiftplants. **a, b**. qRT-PCR analysis of the transcript levels of *OsMBD707* in rice plants transformed with *OsMBD707*-overexpression construct (OX707-#8, OX707-#14, OX707-#20, OX707-#21, OX707-#40) and *OsMBD707*-RNAi construct (707i-#11, 707i-#22, 707i-#26, 707i-#30, 707i-#39), respectively. NPB, nontransgenic wild-type Nipponbare. The rice *Actin*gene was used as an internal control. **c**. Schematic illustration of the *OsMBD707* gene structure and mutations of *OsMBD707 *in CRISPR/Cas9-mediatedmutated plants. Black rectangles represent the 2 exons of *OsMBD707*; Red characters indicate the sequences of the target sites; PAM sequences are underlined; Deletions are indicated by dashes; Numbers on the right side indicate the numbers of deletion nucleotides; WT indicates wild-type sequence

To explore the function of *OsMBD707* in rice, we generated overexpression and RNAi knockdown transgenic plants. For each type, more than 40 independent transgenic T_0_ plants were generated, and five independent plants were chosen for initial analysis. qRT-PCR analysis showed that the transcription levels of *OsMBD707 *were significantly higher in plants transformed with *OsMBD707*-overexpression construct (about 12- to 43- fold) and lower in plants transformed with *OsMBD707*-RNAi construct (about 11 to 27%), as compared to wild-type plants (Fig. 4a, b), confirming the overexpression and knock-down of *OsMBD707*, respectively, in the transgenic plants. In addition, we generated CRISPR/Cas9 knockout plants of *OsMBD707*. Genotyping of the CRISPR/Cas9 transgenic plants identified nine independent T_0_ plants with homozygous mutations in at least one of the single guide RNA (sgRNA) targeting sites of *OsMBD707*,among which seven carried homozygous frameshift mutations: mbd707-#6, mbd707-#9, mbd707-#12, mbd707-#15, mbd707-#22, mbd707-#25, and mbd707-#28 (Fig. 4c). Initial phenotypic observation showed that the *OsMBD707*-overexpression plants (referred to as OX707) displayed a larger tiller angle after tillering stage compared to wild-type. In contrast, no obvious morphological differences were observed among wild-type, the *OsMBD707*-RNAi plants (referred to as 707i) and the CRISPR/Cas9 edited frameshift plants.

**Fig. 5:**
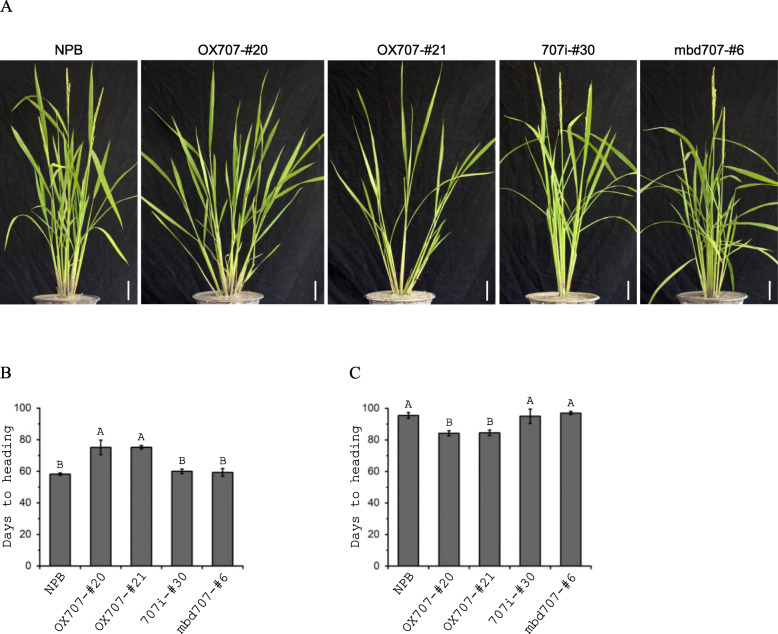
Overexpression of *OsMBD707* causes larger tiller angles and reduced photoperiod sensitivity. **a**. Morphological phenotypes of *OsMBD707*-overexpression (OX707-#20, OX707-#21), -knockdown (707i-#30) and -frameshift (mbd707-#6) plants. NPB, nontransgenic wild-type Nipponbare. **b**. Flowering times of *OsMBD707*-overexpression (OX707-#20, OX707-#21), -knockdown (707i-#30) and -frameshift (mbd707-#6) plants under short day (9.5-h light, 28 °C/14.5-h dark, 26 °C). **c**. Flowering times of *OsMBD707*-overexpression (OX707-#20, OX707-#21), -knockdown (707i-#30) and -frameshift (mbd707-#6) plants under long day (14.5-h light, 28 °C/9.5-h dark, 26 °C). The letters A and B indicate significant differences according to LSD multiple range test at *P* ≤ 0.01

*OsMBD707*-overexpression, -knockdown, and -frameshiftlines were generated up to T_3_ to T_4_ generations, and two independent overexpression lines, one knockdown line, and one frameshift mutant line were chosen for further analysis. Consistent with initial phenotypic observation, the two *OsMBD707*-overexpression lines OX707-#20 and OX707-#21 displayed larger tiller angles, compared to wild-type, the knockdown line 707i-#30, and the frameshift line mbd707-#6 (Fig. 5a). In addition, we observed significant delays in flowering of the two overexpression lines grown under short day (SD) condition (Fig. 5a). We further investigated the heading dates of the *OsMBD707*-overexpression, −knockdown, and -frameshift lines in growth chambers under SD and long day (LD) conditions. As showed in Fig. 5b, under SD, the flowering times of OX707-#20 and OX707-#21 were significantly delayed (about 15–17 days) compared with that of wild-type, 707i-#30 or mbd707-#6(Fig. 5b). In contrast, under LD, the flowering times of OX707-#20 and OX707-#21 were significantly earlier (about 10.5–12.5 days) compared with that of wild-type, 707i-#30 or mbd707-#6 (Fig. 5c), indicating that overexpression of *OsMBD707* caused reduced photoperiod sensitivity in transgenic rice plants.

### Global transcriptome analysis reveals transcriptional changes in key flowering regulator genes induced by overexpression of *MBD707*

RNA-seq-based transcriptome analysis was performed to investigate global transcript changes in the *OsMBD707*-overexpression transgenic line OX707-#21under both SD and LD conditions. Under SD, about 1026 genes were identified that were differentially expressed between OX707-#21 and wild-type, and of these, 616 genes were up-regulated, whereas 410 genes were down-regulated in OX707-#21 (Additional file 4: Figure S2A, Additional file 5: Table S3). Under LD, about 1653 differentially expressed genes (DEGs) were identified between OX707-#21 and wild-type, including 997 up-regulated genes and 656 down-regulated genes in OX707-#21 (Additional file 4: Figure S2A, Additional file 6: Table S4). In total, about 2353 DEGs were identified under SD and/or LD (Additional file 4: Figure S2B).

Gene Ontology (GO) analysis of these 2353 DEGs showed that the significant biological process categories were associated with translation, peptide biosynthetic, peptide metabolic, amide biosynthetic, and cellular amide metabolic processes; the significant cellular component categories were ribosome, ribonucleoprotein complex, non-membrane-bounded organelle, intracellular non-membrane-bounded organelle, and cytoplasmic part; and the significant molecular function categories were involved in structural constituent of ribosome, structural molecule activity, serine-type endopeptidase inhibitor activity, endopeptidase inhibitor activity, peptidase inhibitor activity, peptidase regulator activity, endopeptidase regulator activity, transferase activity/transferring glycosyl groups, N-acetyltransferase activity, N-acyltransferase activity (Additional file 7: Figure S3,Additional file 8: Table S5). Kyoto Encyclopedia of Genes and Genomes (KEGG) analysis revealed that the identified DEGs were significantly enriched in ribosome and phenylpropanoid biosynthesis pathways (Additional file 9: Figure S4, Additional file 10: Table S6).

**Fig. 6:**
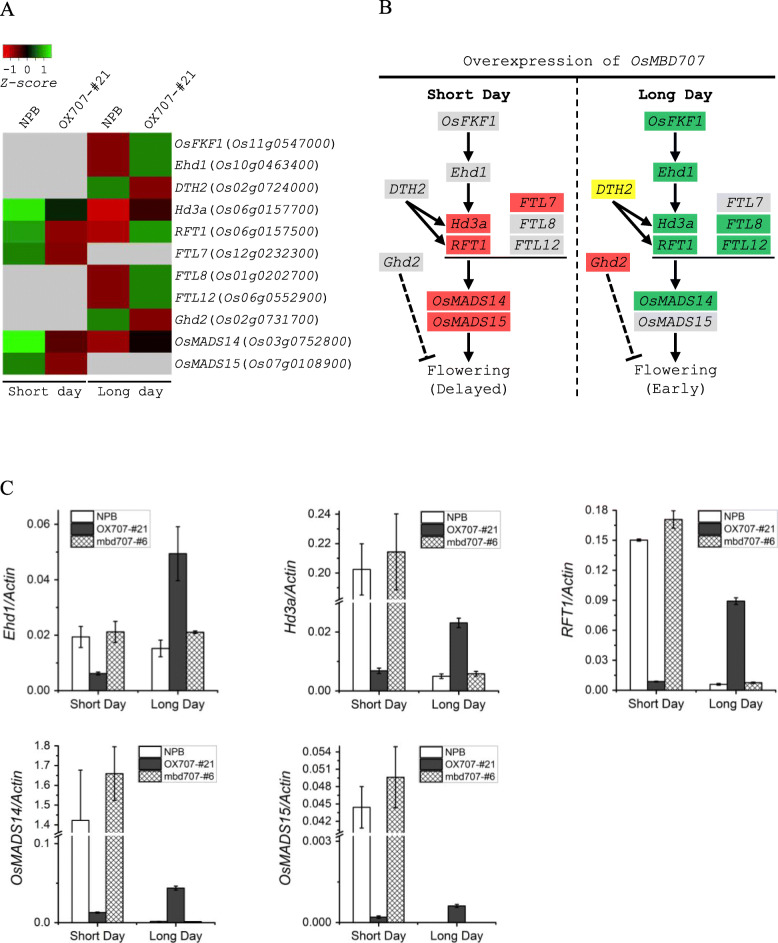
Transcriptional changes of key flowering regulator genes in the *Ehd1-Hd3a*/*RFT1* pathway induced by overexpression of *MBD707*. **a**. Heatmap showing the differential expression levels of flowering genes in the *OsMBD707*-overexpression line (OX707-#21) and wild-type (NPB) revealed by RNA-seq. Grey blocks indicate that the genes were not detected as differentially expressed genes (DEGs) by RNA-seq. **b**. Schematic model showing the differentially expressed flowering genes involved in the *Ehd1-Hd3a*/*RFT1 *regulatory network. Red and green backgrounds indicate that the genes were down-regulated and up-regulated, respectively, in OX707-#21. Yellow background represents that the down-regulation of *DTH2* was paradoxical to the early flowering phenotype of OX707-#21. Grey backgrounds represent that the genes were not detected as DEGs by RNA-seq. Black arrows indicate a promoting effect, bars indicate a repressive effect, and dotted lines indicate an unknown pathway. **c**. qRT-PCR validation of five key flowering regulator genes *Ehd1*, *Hd3a*, *RFT1*, *OsMADS14*, and *OsMADS15* that were identified as DEGs between OX707-#21 and NPB by RNA-seq. The frameshift mutant line mbd707-#6 was also included in the experiment. The rice *Actin* gene was used as an internal control.

We further surveyed the DEGs with known or putative functions involved in tiller angle or flowering time regulation. The phytochrome-interacting factor-like protein gene *OsPIL15 *(*Os01g0286100*) that negatively regulates tiller angle [30] was significantly down-regulated in OX707-#21 under both SD and LD (Additional file 5: Table S3, Additional file 6: Table S4). However, no other known tiller angle regulator genes were identified as DEGs. Notably, a number of genes with functions in controlling flowering time were identified among the DEGs, including *FLAVIN-BINDING, KELCH REPEAT, F-Box 1*(*OsFKF1*) [31], *Early heading date1* (*Ehd1*) [32, 33], *Days to heading on chromosome 2* (*DTH2*) [34], *Heading date3a* (*Hd3a*) and *RICE FLOWERING LOCUS T1 *(*RFT1*) [35, 36], *OsMADS14 *and *OsMADS15* [37], and *Flowering Locus T* gene homologs *FT-L7*, *FT-L8* and *FT-L12* that promote flowering, and *Grain number, plant height, and heading date2* (*GHd2*) [38] that inhibits flowering. Under SD, *Hd3a*, *RFT1*, *FT-L7*, *OsMADS14*, and *OsMADS15* were down-regulated in OX707-#21. In contrast, *OsFKF1*, *Ehd1*, *Hd3a*, *RFT1*, *FT-L8*, *FT-L12*, and *OsMADS14 *were up-regulated, whereas *Ghd2* was down-regulated in OX707-#21 under LD (Fig. 6a, b). The transcriptional changes of these flowering regulator genes in OX707-#21 were consistent with the delayed flowering and early flowering phenotypes of the *MBD707*-overexpression line under SD and LD, respectively, except that the minor-effect heading promoting gene *DTH2* was paradoxically down-regulated under LD (Fig. 6a, b). The transcriptional profiles of five key flowering regulator genes, *Ehd1*, *Hd3a*, *RFT1*, *OsMADS14*, and *OsMADS15* were verified by qRT-PCR, and the results were consistent with the RNA-seq data (Fig. 6c). The frameshift mutant line mbd707-#6 was also included for qRT-PCR, and the expression patterns of the five genes in mbd707-#6 were similar to that in wild-type plant (Fig. 6c).

## Discussion

MBD family proteins have been functionally characterized in various plant species, including *Arabidopsis* [9–23], wheat [24, 25], maize [26], and tomato [27, 28]. MBD proteins play pivotal roles in plant growth, development, and stress responses. In the present study, we characterized OsMBD707 in rice and demonstrated its roles in regulating tiller architecture and flowering time.

Seventeen sequences were predicted as putative MBD proteins from the rice genome (Additional file 1: Table S1). In the present study, 13 predicted *OsMBD* genes were detected to be actively expressed in the roots, stems, leaves, spikeletes, seeds, or panicle axes (Fig. 1). However, no transcripts were detected in any tested tissues for the rest four predicted sequences (Additional file 1: Table S1). The digital expression values of these four sequences were undetectable or almost undetectable in the RGAP database (Additional file 2: Table S2). Whether these four sequences are pseudogenes, or their expression is restricted to other specific tissues or developmental stages, remains to be further elucidated. The plant MBDs were grouped into eight classes, but among them, class IV and class VI were present only in dicots [11]. OsMBD707 belongs to the class I MBD proteins [11]. Previous studies have demonstrated that the *Arabidopsis* class I MBD proteins AtMBD10 and AtMBD11 had important functions in regulating nucleolar dominance [20], and morphological development [9]. RNAi-mediated knockdown of *AtMBD10* revealed that AtMBD10 was required for rRNA gene silencing in nucleolar dominance [20], and knockdown of *AtMBD11* led to aerial rosettes, serrated leaves, abnormal flower position, late flowering, and reduced fertility in *Arabidopsis* [9]. In the present study, we observed that overexpression of *OsMBD707* caused larger tiller angles and reduced photoperiod sensitivity in rice. On the contrary, both knockdown and frameshift mutation of *OsMBD707* did not result in observable changes in the morphology of rice plants. One possible reason might be the functional redundancy between OsMBD707 and other OsMBDs, although they could be bioinformatically grouped into different classes.

Both tiller architecture and flowering time are important traits for rice cultivation. Over the past decades, several key/major regulators of the tiller angle have been identified, such as LAZY1 [39], Tiller Angle Control 1 (TAC1) [40], PROSTRATE GROWTH 1 (PROG1) [41, 42], Loose Plant Architecture 1 (LPA1) [43], PLANT ARCHITECTURE AND YIELD 1 (PAY1) [44], TAC3 and D2 [45], HEAT STRESS TRANSCRIPTION FACTOR 2D (HSFA2D) [46], TILLER INCLINED GROWTH 1 (TIG1) [47], Tiller Angle Control 4 (TAC4) [48], etc. However, RNA-seq analysis did not identify any of these tiller angle regulating genes as DEGs between the *OsMBD707*-overexpression line OX707-#21 and wild-type, except that an *OsPIL15* gene negatively regulating tiller angle [30] was found to be down-regulated in OX707-#21. Since RNA-seq was conducted using RNAs extracted from fully expanded leaves, one reason for rare detection of the key/major tiller angle regulating genes among the DEGs could be that thesegenes were expressed abundantly in tissues involved in tillering (e.g., stems, tiller base, and tiller node), but not expressed or expressed at very low levels in mature leaves [39, 40]. To test this, we performed qRT-PCR to investigate the expression patterns of these known tiller angle regulator genes in mature leaves of OX707-#21 and wild-type. While *HSFA2D* was detected to be expressed highly in mature leaves, no transcripts of *LAZY1*, *PROG1*, *LPA1* and *TIG1*, and very low expression of *TAC1*, *PAY1*, *TAC3*, *D2*, and *TAC4* were detected (Additional file 11: Figure S5). Overall, the expression patterns in mature leaves of these genes, except *OsPIL15 *(Additional file 11: Figure S5B), were not correlated with the larger tiller angle phenotype in *OsMBD707*-overexpression plant. Nevertheless, the mechanism by which OsMBD707 regulates tiller angle remains to be further explored.

Rice is an SD plant whose flowering is promoted under SD, but postponed under LD. Rice flowering time is largely determined by photoperiod sensitivity, which is controlled by an intricate genetic network of an antagonistic regulation of flowering promotion under SD, and repression under LD [49]. In the present study, a number of flowering time genes were identified as DEGs between OX707-#21 and wild-type (Fig. 6a, b). Among these differentially expressed flowering time genes, *Hd3a*, *RFT1*, and *Ehd1* are primary integrators of photoperiodic signals. *Hd3a* and *RFT1* are two florigens of rice and *Ehd1* promotes the expression of *Hd3a* and *RFT1* to induce heading [32, 35, 36]. Via RNA-seq, *Hd3a* and *RFT1* were found to be down-regulated in OX707-#21 under SD, but up-regulated under LD (Fig. 6). *Ehd1* was identified to be up-regulated in OX707-#21 under LD (Fig. 6a), and although RNA-seq did not detect *Ehd1* as a DEG under SD, qRT-PCR verified that *Ehd1* was down-regulated in OX707-#21 under SD (Fig. 6b). Besides, RNA-seq revealed that three flowering-promoting genes/homologs involved in the *Ehd1-Hd3a*/*RFT1* pathway, *OsMADS14*, *OsMADS15*, and *FTL7* were down-regulated in OX707-#21 under SD; and four genes/homologs *OsFKF1*, *OsMADS14*, *FTL8*, and *FTL12* were up-regulated in OX707-#21 under LD (Fig. 6a, b). Although a *DTH2* gene having a function in promoting heading by inducing *Hd3a* and *RFT1* was paradoxically down-regulated in OX707-#21 under LD (Fig. 6a, b), its effect did not seem to change the up-regulation of *Hd3a* and *RFT1*. Overall, RNA-seq results indicated that overexpression of *OsMBD707* led to transcriptional changes in key flowering regulator genes in the *Ehd1-Hd3a*/*RFT1* pathway. In the present study, we observed that *OsMBD707 *itself is expressed at similar levels under SD or LD (Additional file 11: Figure S5C). Our results suggested that overexpression of *OsMBD707 *may cause certain epigenetic changes related to the expression of these key flowering regulator genes. However, we have not explored the detailed connections between OsMBD707 and the flowering regulator genes, the mechanism underlying the role of OsMBD707 in regulating photoperiod sensitivity remains to be further investigated.

## Conclusion

In this study, the bioinformatically predicted *OsMBD* family genes were verified and 13 *OsMBDs* were identified to be actively expressed in various rice tissues. We further performed functional study of OsMBD707, and demonstrated that OsMBD707 is constitutively expressed and localized in the nucleus. Overexpression of *OsMBD707* causes larger tiller angles, delayed flowering under SD and early flowering under LD in rice. RNA-seq analysis revealed that overexpression of *OsMBD707* led to reduced photoperiod sensitivity in rice by down-regulating flowering-promoting genes under SD and up-regulating flowering-promoting genes under LD. Our results suggested the biological roles of OsMBD707 in rice growth and development, and lay the foundation for future studies on the function of OsMBD proteinsin molecular, cellular, and biological processes in rice.

## Methods

### Plant materials and growth conditions

*Oryza sativa* L. ssp. *japonica* (cv. Nipponbare) (maintained in Biotechnology Research Institute, Fujian Academy of Agricultural Sciences) was used in this study. Rice plants were cultured in a greenhouse under partially regulated conditions of 26–32 °C with a 14-h light/10-h dark cycle. For flowering time investigation, rice plants were grown in environmentally controlled growth chambers under SD (9.5-h light, 28 °C/14.5-h dark, 26 °C) and LD (14.5-h light, 28 °C/9.5-h dark, 26 °C) conditions, respectively.

### Gene expression analysis by qRT-PCR

Total RNAs were extracted from rice tissues using TRIzol reagent (Invitrogen, USA). The RNA samples were treated with DNase I (Takara, Dalian, China). The first-strand complementary DNA was generated from 0.5 μg RNA using a ReverTra Ace qPCR RT Kit (TOYOBO, Japan). qRT-PCR was performed on an ABI QuantStudio 6 Flex System (Applied Biosystems, USA) using a FastStart Universal SYBR green Master (ROX) (Roche, Germany). Three replications were conducted for each sample. The primers used for analysis are listed in Additional file 12: Table S7.

### Phylogenetic analysis

The full-length amino acid of OsMBD707 was used as a query to search against the PLAZA database (https://bioinformatics.psb.ugent.be/plaza/). The retrieved sequences were verified against the NCBI non-redundant (NR) protein database (http://blast.ncbi.nlm.nih.gov/). The homologs were aligned using Clustal X program [50], and the phylogenetic tree was constructed using the neighbor-joining algorithm with 1000 bootstrap replicates in MEGA X [51].

### Plasmids construction

A 1935-bp DNA sequence upstream of the ATG start codon of *OsMBD707* was amplified by PCR from Nipponbare genomic DNA. The amplified fragment was cloned into *pCXGUS-P* [52], resulting in a binary vector containing a fusion of the *OsMBD707* promoter and a *GUS* reporter gene. The open reading frame (ORF) of *OsMBD707* was amplified by using specific primers and was cloned into *pCXUN* [52], resulting in a binary vector *pCXUN-OsMBD707* in which *OsMBD707* was driven by the maize ubiquitin-1 promoter. A 213-bp DNA fragment of *OsMBD707* and a 388-bp stuffer DNA fragment from the *GUS* gene were amplified by using specific primers. Overlapping PCR was performed using the two amplified fragments as templates. The resultant hairpin RNAi fragment was cloned into *pCXUN* [52], resulting in a binary RNAi vector *pCXUN-OsMBD707-RNAi*. Two sgRNA sequences targeting the coding region of *OsMBD707* were designed according to Shan’s program [53]. Construction of the CRISPR/Cas9 vector was carried out as previously described [54]. Tow DNA oligos corresponding to the designed sgRNAs were synthesized and the dimer was cloned into *pYLgRNA-OsU6a* and *pYLgRNA-OsU6b*, respectively. The resultant sgRNA expression cassettes were thus cloned into *pYLCRISPR/Cas9Pubi-H* [54]. To generate subcellular localization construct for *OsMBD707*, the ORF of a *GFP* gene was amplified and cloned into *pCXSN* [52], resulting in a binary vector *pCS-NGFP*. The *OsMBD707* ORF digested from *pCXUN-OsMBD707 *by *Bam*HI was then cloned into the *Bam*HI-digested *pCS-NGFP* to fused in-frame with the *GFP* gene. All primers or oligos used for plasmid construction are listed in Additional file 12: Table S7.

### Rice protoplast transfection and stable transformation

Rice protoplasts were prepared from the sheath and stem tissues of 2-week-old etiolated seedlings. The *GFP *alone and *GFP-OsMBD707* fusion constructs were, respectively, co-transfected with a nucleus marker construct *DsRed-OsH2B* [29] into rice protoplasts via a PEG-mediated method as previously described [55]. Rice stable transformation was conducted as previously described [56]. The binary vectors were introduced into *Agrobacterium tumefaciens* EHA105, and the transformant strains were used to transform rice calli of cv. Nipponbare. Homozygous transgenic plants were screened in T_1_ generation derived from self-pollination of T_0_ plants, and were maintained up to T_3_ to T_4_ generations.

### GUS staining and GFP detection

GUS histochemical staining was performed following the procedure described by [57]. Rice tissues were immersed in X-Gluc (Thermo Fisher Scientific, USA) staining solution at 37 °C overnight and were subsequently rinsed in 70% ethanol at room temperature for 1 or 3 days. Pictures were taken with an Olympus SZX12 stereo microscope. The transfected rice protoplasts were incubated at room temperature for 16–20 h. Fluorescence microscopy was carried out on a Leica DMi8 Laser Scanning Confocal microscope (Leica, Germany) with Excitation/emission wavelengths 488/535 nm for green fluorescence.

### RNA-seq analysis

Fully expanded leaves of the overexpression line OX707-#21 and wild-type were collected at about 50 days after sowing. Total RNAs extracted from leaf tissues were subjected to RNA-seq analysis at Novogene (Beijing, China). Three to four biological replicates of each sample were used for RNA-seq analysis. Briefly, sequencing libraries were generated using NEB Next UltraTM RNA Library Prep Kit for Illumina (NEB, USA) following manufacturer’s instructions. The libraries were sequenced on an Illumina Novaseq platform. Raw reads were processed through in-house perl scripts, and the filtered clean reads were mapped to the reference genome using Hisat2 v2.0.5. FPKM value of each gene was calculated for estimating gene expression levels [58]. All gene count and FPKM values are available in Additional file 13: Table S8. Gene expression difference between OX707-#21 and wild-type was performed using the DESeq2 [59]. Genes with padj< 0.05 and log2FoldChange > 1 were assigned as differentially expressed. GO and KEGG enrichment analysis of DEGs were implemented by the cluster Profiler [60].

## Supplementary Information

**Additional file 1: Table S1**. Methyl-CpG-binding domain protein genes predicted in rice genome.

**Additional file 2: Table S2.** Digital expression profiles of rice Methyl-CpG-binding domain protein genes.

**Additional file 3: Figure S1**. Detection of *OsMBD707* transcript.(A) Schematic diagrams of predicted *OsMBD707* splicing variants. (B) Sequence alignment of predicted *OsMBD707* splicing variants. Sequences for primers designed to distinguish the two predicted alternative transcripts (predicted amplification sizes of 106 bp and 94 bp for *XM_015764399.1/LOC_Os12g42550.1* and *XM_015764400.2/LOC_Os12g42550.2*, respectively.) are indicated by arrow. (C) RT-PCR analysis for detecting predicted *OsMBD707* splicing variants. Only an amplification size of 106 bp for *XM_015764399.1/LOC_Os12g42550.1* was detected in the roots, stems, leaves, spikelets, seeds, and panicle axes. (D) Sequencing confirmation of the 106 bp-amplified product of *XM_015764399.1/LOC_Os12g42550.1*. (PPT 2129 kb)

**Additional file 4: Figure S2**. Summary of differentially expressed genes (DEGs) identified between the *OsMBD707*-overexpression line OX707-#21 and wild-type. (A) Number of DEGs between OX707-#21 and wild-type under short day (SD) and long day (LD) conditions, respectively. Up, up-regulated in OX707-#21; Down, down-regulated in OX707-#21. (B) Venn diagrams of DEGs between OX707-#21 and wild-type under SD and LD. (PPT 128 kb)

**Additional file 5: Table S3**. DEGs between the *OsMBD707*-overexpression line OX707-#21 and wild-type under short day condition. (XLS 314 kb)

**Additional file 6: Table S4**. DEGs between the *OsMBD707*-overexpression line OX707-#21 and wild-type under long day condition. (XLS 486 kb)

**Additional file 7: Figure S3**. GO analysis of DEGs between the *OsMBD707*-overexpression line OX707-#21 and wild-type. (PPT 758 kb)

**Additional file 8: Table S5**. Significant categories identified by GO analysis of the 2353 DEGs between the *OsMBD707*-overexpression line OX707-#21 and wild-type. (XLS 42 kb)

**Additional file 9: Figure S4**. KEGG analysis of DEGs between the *OsMBD707*-overexpression line OX707-#21 and wild-type. (PPT 374 kb)

**Additional file 10: Table S6**. Significant pathways identified by KEGG analysis of the 2353 DEGs between the *OsMBD707*-overexpression line OX707-#21 and wild-type. (XLS 29 kb)

**Additional file 11: Figure S5**. Expression profiles of tiller angle regulator genes in *OsMBD707*-overexpression plant, and of *OsMBD707* under short day (SD) and long day (LD). (PPT 2596 kb)

**Additional file 12: Table S7**. Primers used in this study.

**Additional file 13: Table S8**. Gene count and gene FPKM values from the RNA-seq in this study.

## Data Availability

Raw data was deposited in NCBI database under SRA accession: SRP303927 (https://www.ncbi.nlm.nih.gov/sra/SRP303927).

## Abbreviations

MBD: Methyl-CpG-binding domain
NCBI: National Center for Biotechnology Information
RGAP: Rice Genome Annotation Project
FPKM: Expected Fragments PerKilobase of transcript per Million fragments sequenced
DAP: Days after pollination
qRT-PCR: Quantitative RT-PCR
SD: Short day
LD: Long day
DEGs: Differentially expressed genes
GO: Gene Ontology
KEGG: Kyoto Encyclopedia of Genes and Genomes
ORF: Open reading frame
sgRNA: Single guide RNA
