# Supplementary material for: Overexpression of a methyl-CpG-binding protein gene OsMBD707 leads to larger tiller angles and reduced photoperiod sensitivity in rice
Source: BMC Plant Biol. 2021 Feb 18;21:100. doi: 10.1186/s12870-021-02880-3 (PMC7893954; doi:10.1186/s12870-021-02880-3)
Supplement: Supplementary file 1 — Additional file 1: Table S1. Methyl-CpG-binding domain protein genes predicted in rice genome. [file 12870_2021_2880_MOESM1_ESM.docx]

**Additional file 1: Table S1.** Methyl-CpG-binding domain protein genes predicted in rice genome

| No. | Gene^a^ | BAC Accession^a^ | cDNA Accession^a^ | Chromosome | Gene ID | |
| --- | --- | --- | --- | --- | --- | --- |
|  |  |  |  |  | RAP Locus | MSU RGAP Locus |
| 1 | MBD701 | AC099403 | AK101317 | 9 | *Os09g0494300* | *LOC_Os09g32090* |
| 2 | MBD703^b^ | AP003726 | CB097036 | 6 | *Os06g0702100* | *LOC_Os06g48870* |
| 3 | MBD704 | AL663008 | CA759629 | 4 | *Os04g0273900* | *LOC_Os04g20560* |
| 4 | MBD705 | AL731611 | AK065632 | [4](https://www.ncbi.nlm.nih.gov/protein/XP_015636705.1) | *Os04g0266400* | *LOC_Os04g19684* |
| 5 | MBD706 | AC104708 | AU057642 | 5 | *Os05g0404600* | *LOC_Os05g33550* |
| 6 | MBD707 | AL732532 | AK059006 | 12 | *Os12g0620400* | *LOC_Os12g42550* |
| 7 | MBD708^c^ | AP006169 | AK058514 | 9 | *Os09g0473350* | *LOC_Os09g29750* |
| 8 | MBD709 | AP004376 | AK069097 | 8 | *Os08g0485700* | *LOC_Os08g37920* |
| 9 | MBD710^d^ | AP005785 | AK072184 | 2 | *Os02g0192400* | *LOC_Os02g09920* |
| 10 | MBD711 | AC104708 | None | 5 | *Os05g0404700* | *LOC_Os05g33554* |
| 11 | MBD712 | AL662965 | None | ‒ | ‒ | ‒ |
| 12 | MBD713^b^ | AL606989 | None | 4 | *Os04g0193900* | *LOC_Os04g11730* |
| 13 | MBD714 | AL606989 | None | 4 | *Os04g0193200* | *LOC_Os04g11640* |
| 14 | MBD715 | AP005918 | AK068243 | 8 | *Os08g0485600* | *LOC_Os08g37904* |
| 15 | MBD716 | AL606989 | None | ‒ | ‒ | ‒ |
| 16 | MBD717 | AL731610 | AK072038 | 4 | *Os04g0613800* | *LOC_Os04g52380* |
| 17 | MBD718 | AP003875 | AK071442 | 8 | *Os08g0206700* | *LOC_Os08g10580* |
| 18 |  |  |  | 4 | *Os04g0192775*^b^ | ‒ |
| 19 |  |  |  | 4 | ‒ | *LOC_Os04g11510*^b^ |

^a^According to Springer and Kaeppler, 2005; ^b^No transcript detected by qRT-PCR; ^c^Overall sequence similarity to MBD8; ^d^Containing a MBD9 motif1; ‒, None RAP Locus or MSU RGAP Locus matched.
