# Supplementary material for: Overexpression of a methyl-CpG-binding protein gene OsMBD707 leads to larger tiller angles and reduced photoperiod sensitivity in rice
Source: BMC Plant Biol. 2021 Feb 18;21:100. doi: 10.1186/s12870-021-02880-3 (PMC7893954; doi:10.1186/s12870-021-02880-3)
Supplement: Supplementary file 2 — Additional file 2: Table S2. Digital expression profiles of rice Methyl-CpG-binding domain protein genes. [file 12870_2021_2880_MOESM2_ESM.docx]

**Additional file 2: Table S2.** Digital expression profiles of rice Methyl-CpG-binding domain protein genes

| Tissue | Rice Genotype | FPKM^a^ | | | | | |
| --- | --- | --- | --- | --- | --- | --- | --- |
|  |  | *MBD701/ Os09g0494300/*  *LOC_Os09g32090* | *MBD703/ Os06g0702100/*  *LOC_Os06g48870* | *MBD704/ Os04g0273900/*  *LOC_Os04g20560* | *MBD705/ Os04g0266400/*  *LOC_Os04g19684* | *MBD706/ Os05g0404600/*  *LOC_Os05g33550* | *MBD707/ Os12g0620400/*  *LOC_Os12g42550* |
| Leaves-20 days | NPB | 14.6582 | 0 | 0 | 17.6347 | 5.48747 | 231.603 |
| Post-emergence inflorescence | NPB | 20.4188 | 0 | 0 | 15.679 | 24.7373 | 146.681 |
| Pre-emergence inflorescence | NPB | 30.8298 | 0 | 0.464855 | 13.9639 | 20.9391 | 128.313 |
| Anther | NPB | 15.7537 | 0 | 0.772486 | 4.84719 | 18.6415 | 52.2989 |
| Pistil | NPB | 36.662 | 0 | 0 | 7.96735 | 32.9258 | 129.534 |
| Seed-5 DAP | NPB | 18.715 | 0 | 0.920414 | 10.2051 | 14.5496 | 32.8797 |
| Embryo-25 DAP | NPB | 35.6139 | 0 | 0 | 16.5331 | 5.40075 | 79.6871 |
| Endosperm-25 DAP | NPB | 5.82286 | 0 | 0 | 1.65767 | 11.7455 | 13.811 |
| Seed-10 DAP | NPB | 8.74523 | 0 | 0 | 4.4349 | 7.39263 | 12.0981 |
| Endosperm-25 DAP (replicate) | NPB | 6.10764 | 0 | 0 | 1.64906 | 12.9307 | 13.806 |
| Leaves-20 days (replicate) | NPB | 12.9028 | 0 | 0 | 14.9819 | 5.25551 | 180.914 |
| Shoots | NPB | 16.5332 | 0 | 0 | 24.1321 | 6.09588 | 129.177 |
| Seedling |  | 13.6745 | 0 | 0.537761 | 33.695 | 8.6074 | 178.62 |
| Callus |  | 22.5285 | 0.0088 | 0.0285643 | 24.972 | 11.1911 | 82.2965 |
| Panicles |  | 51.3077 | 0 | 0.373358 | 7.53593 | 108.279 | 291.619 |

^a^FPKM (expected Fragments Per Kilobase of transcript per Million fragments sequenced) values were derived from the Rice Genome Annotation Project Database (<http://rice.plantbiology.msu.edu/>); ^b^DAP, days after pollination.

**Additional file 2: Table S2.** Digital expression profiles of rice Methyl-CpG-binding domain protein genes (Continued)

| Tissue | Rice Genotype | FPKM^a^ | | | | | |
| --- | --- | --- | --- | --- | --- | --- | --- |
|  |  | *MBD708/ Os09g0473350/*  *LOC_Os09g29750* | *NBD709/ Os08g0485700/*  *LOC_Os08g37920* | *MBD710/ Os02g0192400/*  *LOC_Os02g09920* | *MBD711/ Os05g0404700/*  *LOC_Os05g33554* | *MBD713/ Os04g0193900/*  *LOC_Os04g11730* | *MBD714/ Os04g0193200/*  *LOC_Os04g11640* |
| Leaves-20 days | NPB | 5.84985 | 7.94069 | 11.4821 | 0 | 0 | 0 |
| Post-emergence inflorescence | NPB | 16.7324 | 10.8398 | 12.416 | 3.54548 | 0 | 0 |
| Pre-emergence inflorescence | NPB | 20.4963 | 12.7448 | 17.6305 | 0 | 0 | 0 |
| Anther | NPB | 8.64221 | 3.08658 | 9.66931 | 0 | 0 | 0.8462 |
| Pistil | NPB | 28.1562 | 28.5947 | 18.7009 | 0 | 0 | 0 |
| Seed-5 DAP | NPB | 9.55399 | 12.5561 | 28.8228 | 66.6043 | 0.8691 | 0 |
| Embryo-25 DAP | NPB | 19.7865 | 49.7222 | 15.5807 | 2.83318 | 0 | 0 |
| Endosperm-25 DAP | NPB | 2.99546 | 3.36864 | 9.34565 | 56.1541 | 0 | 0 |
| Seed-10 DAP | NPB | 5.41901 | 5.45778 | 24.9896 | 74.2708 | 0 | 0 |
| Endosperm-25 DAP (replicate) | NPB | 3.07399 | 3.24869 | 9.83256 | 49.6043 | 0 | 0 |
| Leaves-20 days (replicate) | NPB | 6.55574 | 9.94122 | 9.36438 | 0 | 0 | 0 |
| Shoots | NPB | 4.05276 | 9.40425 | 4.15562 | 0 | 0 | 0 |
| Seedling |  | 5.72857 | 7.53526 | 6.69528 | 0 | 0 | 0 |
| Callus |  | 20.2009 | 29.5385 | 15.9152 | 3.53155 | 0 | 0 |
| Panicles |  | 12.4658 | 8.44491 | 2.27595 | 5.02534 | 0 | 0 |

^a^FPKM (expected Fragments Per Kilobase of transcript per Million fragments sequenced) values were derived from the Rice Genome Annotation Project Database (<http://rice.plantbiology.msu.edu/>); ^b^DAP, days after pollination.

**Additional file 2: Table S2.** Digital expression profiles of rice Methyl-CpG-binding domain protein genes (Continued)

| Tissue | Rice Genotype | FPKM^a^ | | | | | |
| --- | --- | --- | --- | --- | --- | --- | --- |
|  |  | *MBD715/ Os08g0485600/*  *LOC_Os08g37904* | *NBD717/ Os04g0613800/*  *LOC_Os04g52380* | *MBD718/ Os08g0206700/*  *LOC_Os08g10580* | *Os04g0192775* | *LOC_Os04g11510* |  |
| Leaves-20 days | NPB | 5.27782 | 12.3866 | 6.68532 | ‒ | 0 |  |
| Post-emergence inflorescence | NPB | 8.06354 | 27.0087 | 5.03602 | ‒ | 0 |  |
| Pre-emergence inflorescence | NPB | 10.1458 | 16.5226 | 8.86486 | ‒ | 0 |  |
| Anther | NPB | 3.35755 | 5.61167 | 3.65698 | ‒ | 0 |  |
| Pistil | NPB | 15.7456 | 32.5563 | 4.60256 | ‒ | 0 |  |
| Seed-5 DAP^b^ | NPB | 5.27057 | 6.35819 | 3.24772 | ‒ | 0 |  |
| Embryo-25 DAP | NPB | 14.2756 | 21.746 | 6.50513 | ‒ | 0 |  |
| Endosperm-25 DAP | NPB | 2.41522 | 0.804361 | 2.43162 | ‒ | 0 |  |
| Seed-10 DAP | NPB | 4.2568 | 1.36532 | 5.87719 | ‒ | 0 |  |
| Endosperm-25 DAP (replicate) | NPB | 2.46036 | 0.881177 | 2.49724 | ‒ | 0 |  |
| Leaves-20 days (replicate) | NPB | 6.41858 | 13.1793 | 7.45226 | ‒ | 0 |  |
| Shoots | NPB | 9.35485 | 17.0381 | 9.42187 | ‒ | 0 |  |
| Seedling |  | 5.45839 | 14.9025 | 7.83779 | ‒ | 0 |  |
| Callus |  | 10.5351 | 13.819 | 14.1749 | ‒ | 0 |  |
| Panicles |  | 3.90124 | 31.1632 | 3.11935 | ‒ | 0 |  |

^a^FPKM (expected Fragments Per Kilobase of transcript per Million fragments sequenced) values were derived from the Rice Genome Annotation Project Database (<http://rice.plantbiology.msu.edu/>); ^b^DAP, days after pollination; ‒, no hits found in the database.
