# Supplementary material for: Overexpression of a methyl-CpG-binding protein gene OsMBD707 leads to larger tiller angles and reduced photoperiod sensitivity in rice
Source: BMC Plant Biol. 2021 Feb 18;21:100. doi: 10.1186/s12870-021-02880-3 (PMC7893954; doi:10.1186/s12870-021-02880-3)
Supplement: Supplementary file 3 — Additional file 3: Figure S1. Detection of OsMBD707 transcript.(A) Schematic diagrams of predicted OsMBD707 splicing variants. (B) Sequence alignment of predicted OsMBD707 splicing variants. Sequences for primers designed to distinguish the two predicted alternative transcripts (predicted amplification sizes of 106 bp and 94 bp for XM_015764399.1/LOC_Os12g42550.1 and XM_015764400.2/LOC_Os12g42550.2, respectively.) are indicated by arrow. (C) RT-PCR analysis for detecting predicted OsMBD707 splicing variants. Only an amplification size of 106 bp for XM_015764399.1/LOC_Os12g42550.1 was detected in the roots, stems, leaves, spikelets, seeds, and panicle axes. (D) Sequencing confirmation of the 106 bp-amplified product of XM_015764399.1/LOC_Os12g42550.1. (PPT 2129 kb) [file 12870_2021_2880_MOESM3_ESM.ppt]

## Slide 1
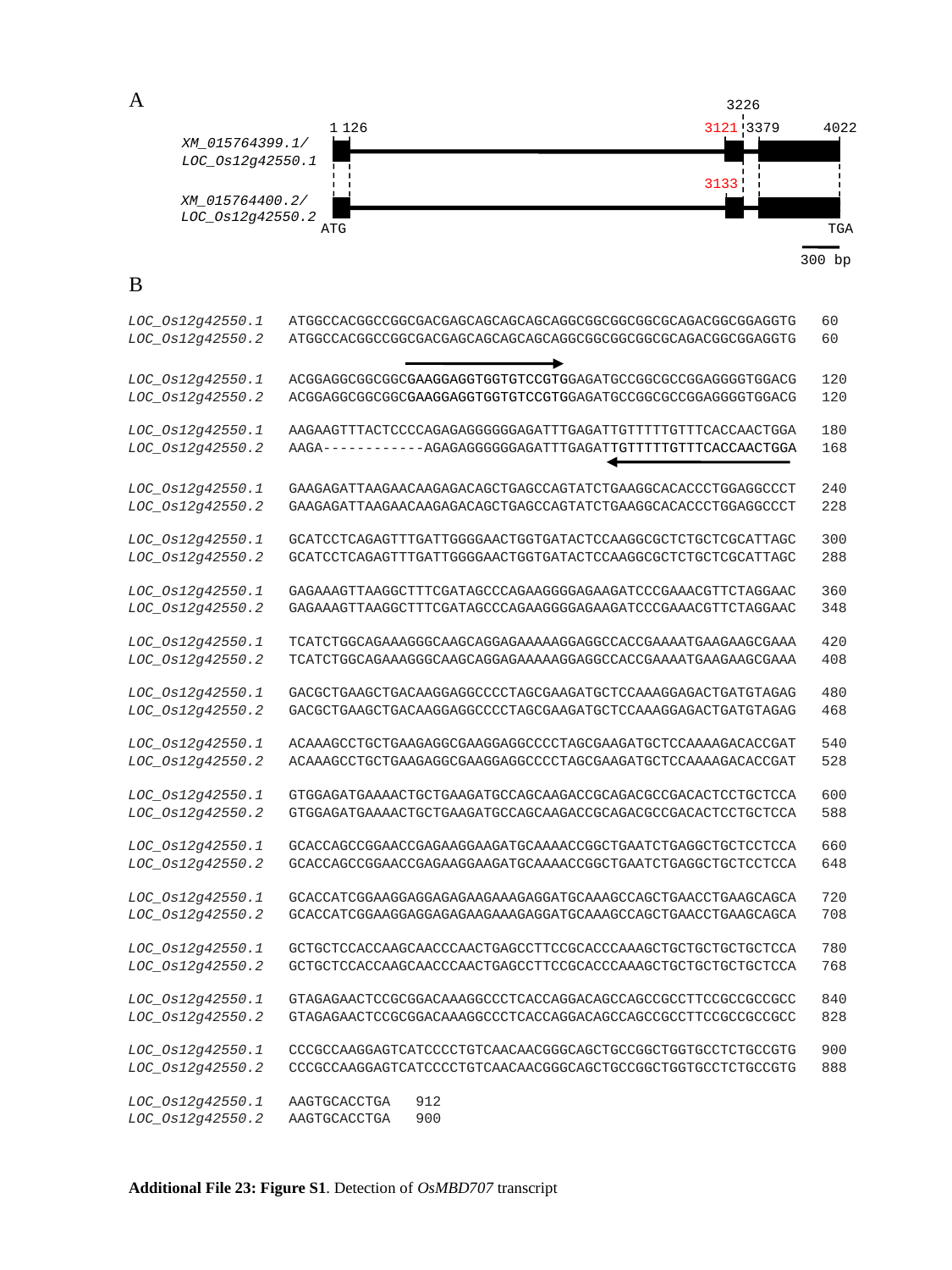

A
3226
126
3121
3379
4022
1
XM_015764399.1/
LOC_Os12g42550.1
3133
XM_015764400.2/
LOC_Os12g42550.2
ATG
TGA
300 bp
B
LOC_Os12g42550.1 ATGGCCACGGCCGGCGACGAGCAGCAGCAGCAGGCGGCGGCGGCGCAGACGGCGGAGGTG 60
LOC_Os12g42550.2 ATGGCCACGGCCGGCGACGAGCAGCAGCAGCAGGCGGCGGCGGCGCAGACGGCGGAGGTG 60
LOC_Os12g42550.1 ACGGAGGCGGCGGCGAAGGAGGTGGTGTCCGTGGAGATGCCGGCGCCGGAGGGGTGGACG 120
LOC_Os12g42550.2 ACGGAGGCGGCGGCGAAGGAGGTGGTGTCCGTGGAGATGCCGGCGCCGGAGGGGTGGACG 120
LOC_Os12g42550.1 AAGAAGTTTACTCCCCAGAGAGGGGGGAGATTTGAGATTGTTTTTGTTTCACCAACTGGA 180
LOC_Os12g42550.2 AAGA------------AGAGAGGGGGGAGATTTGAGATTGTTTTTGTTTCACCAACTGGA 168
LOC_Os12g42550.1 GAAGAGATTAAGAACAAGAGACAGCTGAGCCAGTATCTGAAGGCACACCCTGGAGGCCCT 240
LOC_Os12g42550.2 GAAGAGATTAAGAACAAGAGACAGCTGAGCCAGTATCTGAAGGCACACCCTGGAGGCCCT 228
LOC_Os12g42550.1 GCATCCTCAGAGTTTGATTGGGGAACTGGTGATACTCCAAGGCGCTCTGCTCGCATTAGC 300
LOC_Os12g42550.2 GCATCCTCAGAGTTTGATTGGGGAACTGGTGATACTCCAAGGCGCTCTGCTCGCATTAGC 288
LOC_Os12g42550.1 GAGAAAGTTAAGGCTTTCGATAGCCCAGAAGGGGAGAAGATCCCGAAACGTTCTAGGAAC 360
LOC_Os12g42550.2 GAGAAAGTTAAGGCTTTCGATAGCCCAGAAGGGGAGAAGATCCCGAAACGTTCTAGGAAC 348
LOC_Os12g42550.1 TCATCTGGCAGAAAGGGCAAGCAGGAGAAAAAGGAGGCCACCGAAAATGAAGAAGCGAAA 420
LOC_Os12g42550.2 TCATCTGGCAGAAAGGGCAAGCAGGAGAAAAAGGAGGCCACCGAAAATGAAGAAGCGAAA 408
LOC_Os12g42550.1 GACGCTGAAGCTGACAAGGAGGCCCCTAGCGAAGATGCTCCAAAGGAGACTGATGTAGAG 480
LOC_Os12g42550.2 GACGCTGAAGCTGACAAGGAGGCCCCTAGCGAAGATGCTCCAAAGGAGACTGATGTAGAG 468
LOC_Os12g42550.1 ACAAAGCCTGCTGAAGAGGCGAAGGAGGCCCCTAGCGAAGATGCTCCAAAAGACACCGAT 540
LOC_Os12g42550.2 ACAAAGCCTGCTGAAGAGGCGAAGGAGGCCCCTAGCGAAGATGCTCCAAAAGACACCGAT 528
LOC_Os12g42550.1 GTGGAGATGAAAACTGCTGAAGATGCCAGCAAGACCGCAGACGCCGACACTCCTGCTCCA 600
LOC_Os12g42550.2 GTGGAGATGAAAACTGCTGAAGATGCCAGCAAGACCGCAGACGCCGACACTCCTGCTCCA 588
LOC_Os12g42550.1 GCACCAGCCGGAACCGAGAAGGAAGATGCAAAACCGGCTGAATCTGAGGCTGCTCCTCCA 660
LOC_Os12g42550.2 GCACCAGCCGGAACCGAGAAGGAAGATGCAAAACCGGCTGAATCTGAGGCTGCTCCTCCA 648
LOC_Os12g42550.1 GCACCATCGGAAGGAGGAGAGAAGAAAGAGGATGCAAAGCCAGCTGAACCTGAAGCAGCA 720
LOC_Os12g42550.2 GCACCATCGGAAGGAGGAGAGAAGAAAGAGGATGCAAAGCCAGCTGAACCTGAAGCAGCA 708
LOC_Os12g42550.1 GCTGCTCCACCAAGCAACCCAACTGAGCCTTCCGCACCCAAAGCTGCTGCTGCTGCTCCA 780
LOC_Os12g42550.2 GCTGCTCCACCAAGCAACCCAACTGAGCCTTCCGCACCCAAAGCTGCTGCTGCTGCTCCA 768
LOC_Os12g42550.1 GTAGAGAACTCCGCGGACAAAGGCCCTCACCAGGACAGCCAGCCGCCTTCCGCCGCCGCC 840
LOC_Os12g42550.2 GTAGAGAACTCCGCGGACAAAGGCCCTCACCAGGACAGCCAGCCGCCTTCCGCCGCCGCC 828
LOC_Os12g42550.1 CCCGCCAAGGAGTCATCCCCTGTCAACAACGGGCAGCTGCCGGCTGGTGCCTCTGCCGTG 900
LOC_Os12g42550.2 CCCGCCAAGGAGTCATCCCCTGTCAACAACGGGCAGCTGCCGGCTGGTGCCTCTGCCGTG 888
LOC_Os12g42550.1 AAGTGCACCTGA 912
LOC_Os12g42550.2 AAGTGCACCTGA 900
Additional File 23: Figure S1. Detection of OsMBD707 transcript

## Slide 2
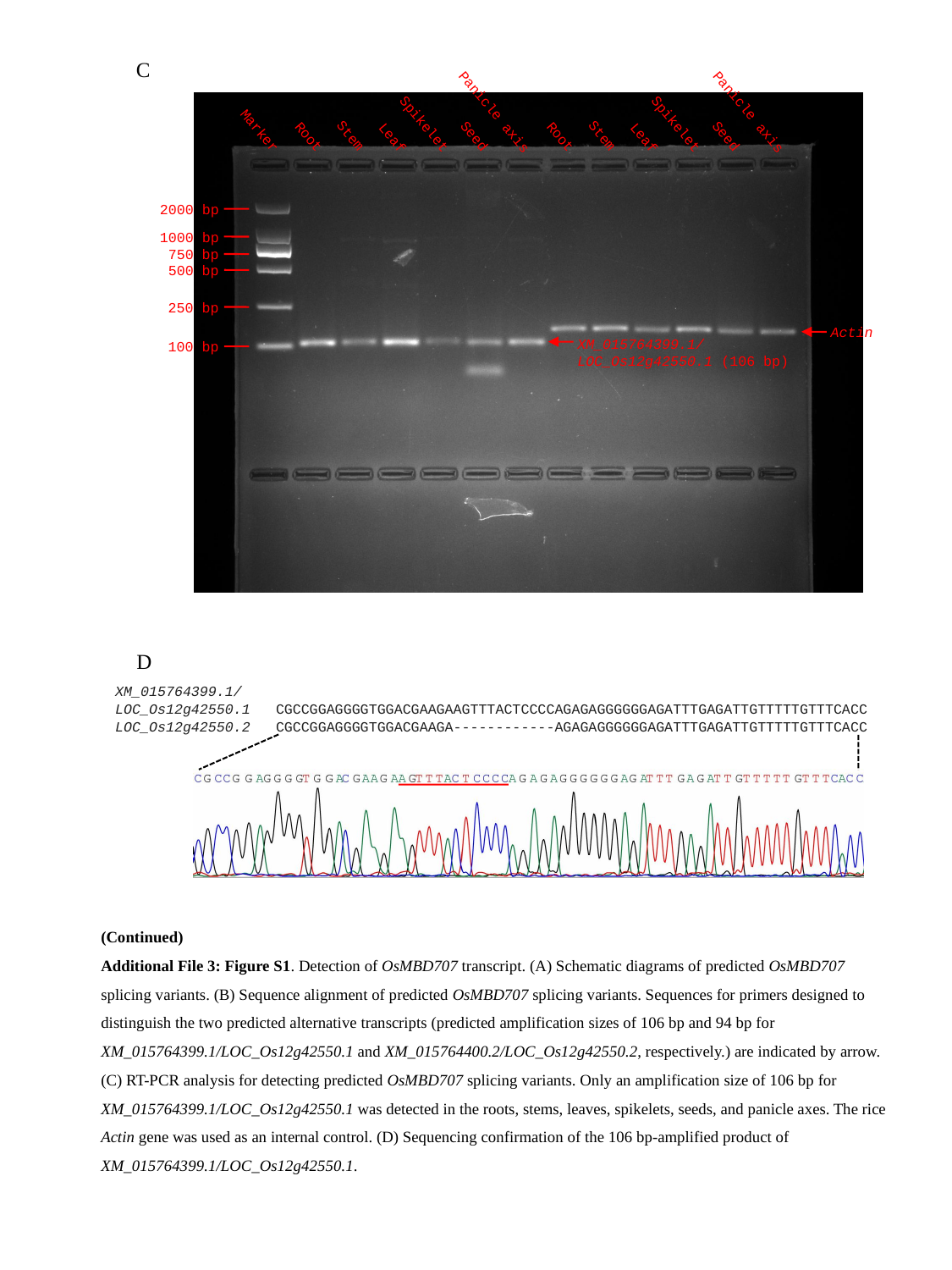

C
Panicle axis
Panicle axis
Spikelet
Spikelet
Marker
Stem
Stem
Seed
Seed
Root
Root
Leaf
Leaf
2000 bp
1000 bp
750 bp
500 bp
250 bp
Actin
XM_015764399.1/
LOC_Os12g42550.1 (106 bp)
100 bp
D
XM_015764399.1/
LOC_Os12g42550.1 CGCCGGAGGGGTGGACGAAGAAGTTTACTCCCCAGAGAGGGGGGAGATTTGAGATTGTTTTTGTTTCACC
LOC_Os12g42550.2 CGCCGGAGGGGTGGACGAAGA------------AGAGAGGGGGGAGATTTGAGATTGTTTTTGTTTCACC
(Continued)
Additional File 3: Figure S1. Detection of OsMBD707 transcript. (A) Schematic diagrams of predicted OsMBD707 splicing variants. (B) Sequence alignment of predicted OsMBD707 splicing variants. Sequences for primers designed to distinguish the two predicted alternative transcripts (predicted amplification sizes of 106 bp and 94 bp for XM_015764399.1/LOC_Os12g42550.1 and XM_015764400.2/LOC_Os12g42550.2, respectively.) are indicated by arrow. (C) RT-PCR analysis for detecting predicted OsMBD707 splicing variants. Only an amplification size of 106 bp for XM_015764399.1/LOC_Os12g42550.1 was detected in the roots, stems, leaves, spikelets, seeds, and panicle axes. The rice Actin gene was used as an internal control. (D) Sequencing confirmation of the 106 bp-amplified product of XM_015764399.1/LOC_Os12g42550.1.
