# Supplementary material for: Overexpression of a methyl-CpG-binding protein gene OsMBD707 leads to larger tiller angles and reduced photoperiod sensitivity in rice
Source: BMC Plant Biol. 2021 Feb 18;21:100. doi: 10.1186/s12870-021-02880-3 (PMC7893954; doi:10.1186/s12870-021-02880-3)
Supplement: Supplementary file 4 — Additional file 4: Figure S2. Summary of differentially expressed genes (DEGs) identified between the OsMBD707-overexpression line OX707-#21 and wild-type. (A) Number of DEGs between OX707-#21 and wild-type under short day (SD) and long day (LD) conditions, respectively. Up, up-regulated in OX707-#21; Down, down-regulated in OX707-#21. (B) Venn diagrams of DEGs between OX707-#21 and wild-type under SD and LD. (PPT 128 kb) [file 12870_2021_2880_MOESM4_ESM.ppt]

## Slide 1
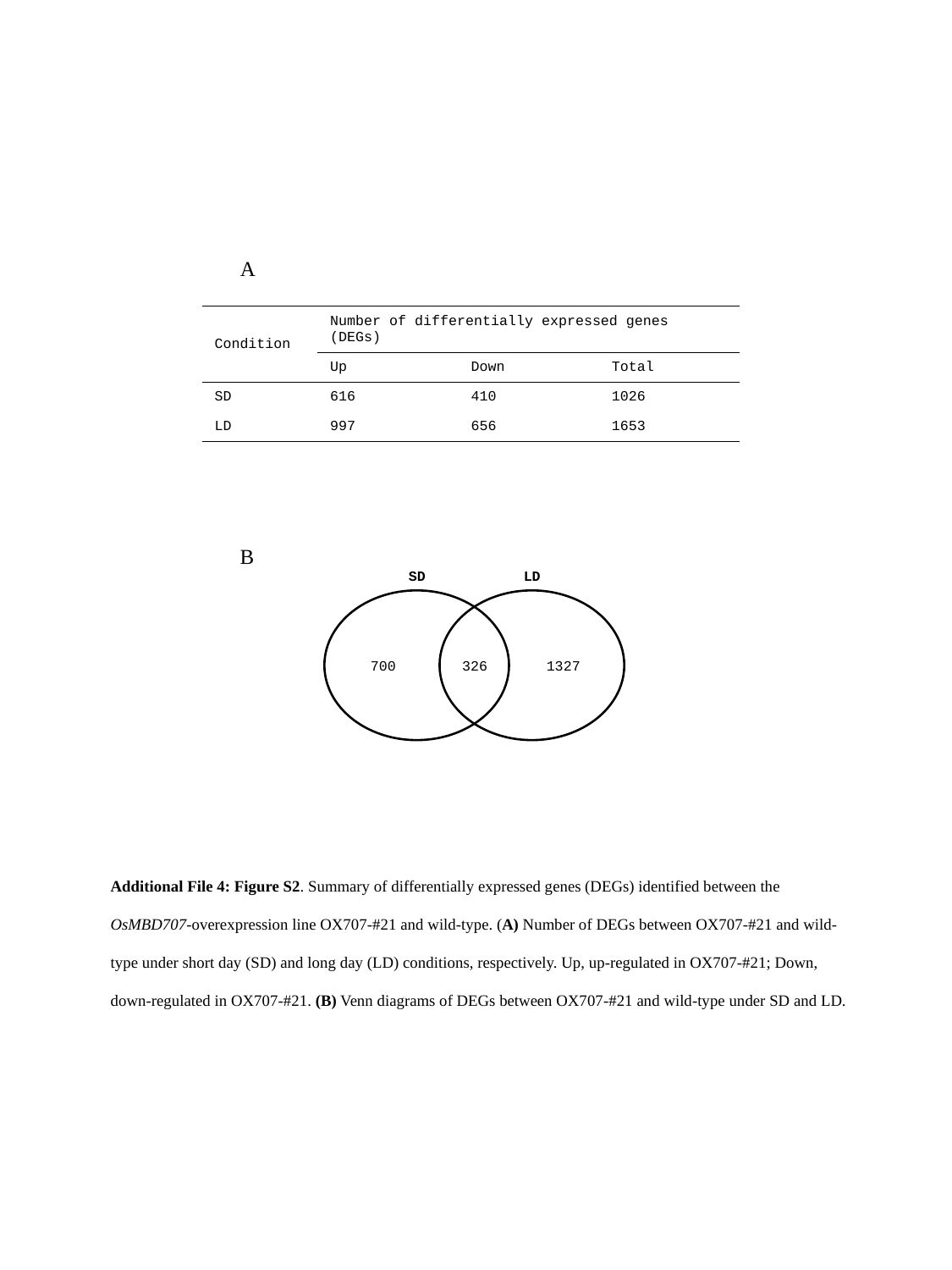

A
| Condition | Number of differentially expressed genes (DEGs) | | |
| --- | --- | --- | --- |
| | Up | Down | Total |
| SD | 616 | 410 | 1026 |
| LD | 997 | 656 | 1653 |
B
LD
SD
326
1327
700
Additional File 4: Figure S2. Summary of differentially expressed genes (DEGs) identified between the OsMBD707-overexpression line OX707-#21 and wild-type. (A) Number of DEGs between OX707-#21 and wild-type under short day (SD) and long day (LD) conditions, respectively. Up, up-regulated in OX707-#21; Down, down-regulated in OX707-#21. (B) Venn diagrams of DEGs between OX707-#21 and wild-type under SD and LD.
