# Supplementary material for: Overexpression of a methyl-CpG-binding protein gene OsMBD707 leads to larger tiller angles and reduced photoperiod sensitivity in rice
Source: BMC Plant Biol. 2021 Feb 18;21:100. doi: 10.1186/s12870-021-02880-3 (PMC7893954; doi:10.1186/s12870-021-02880-3)
Supplement: Supplementary file 7 — Additional file 7: Figure S3. GO analysis of DEGs between the OsMBD707-overexpression line OX707-#21 and wild-type. (PPT 758 kb) [file 12870_2021_2880_MOESM7_ESM.ppt]

## Slide 1
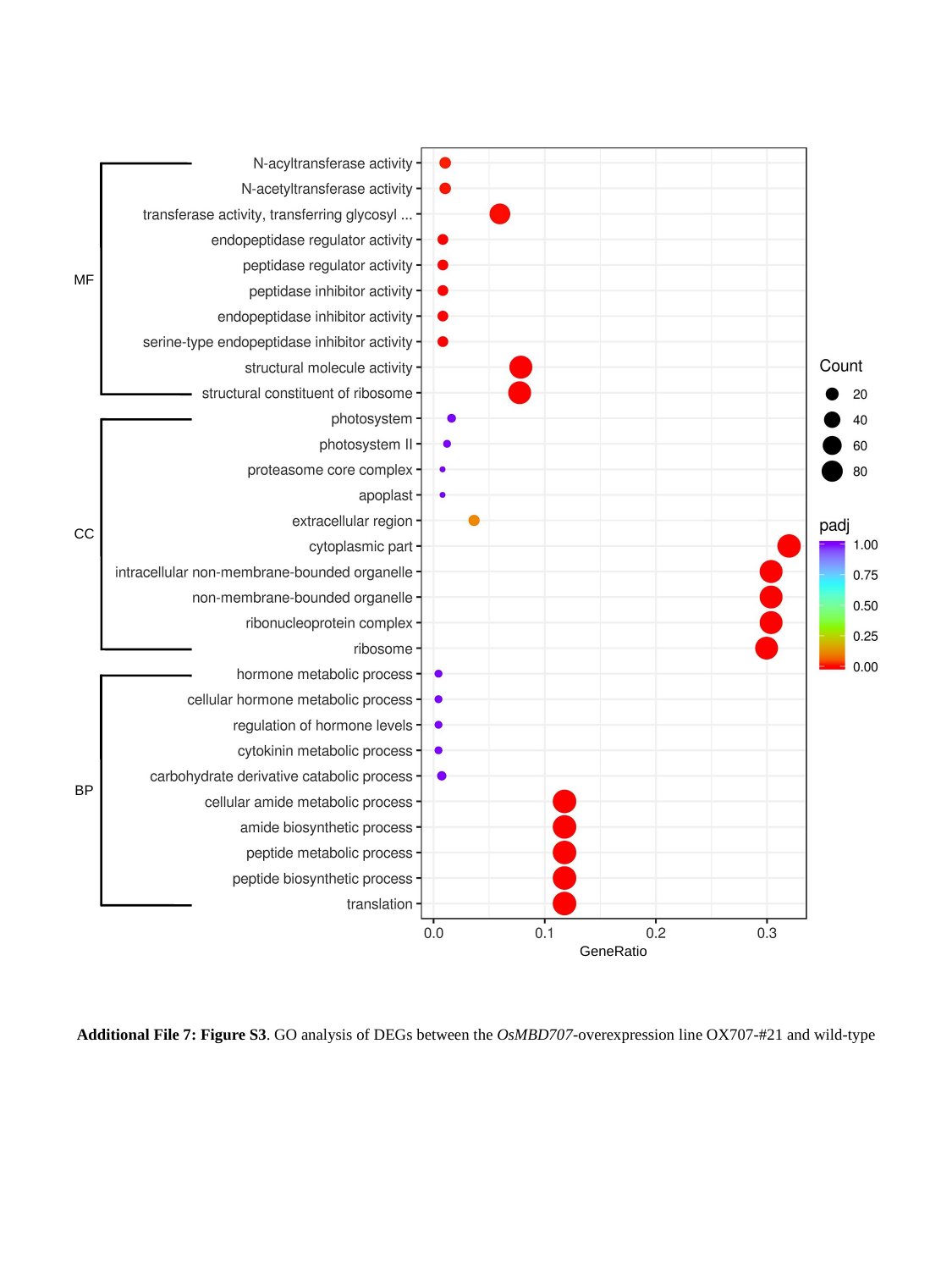

MF
CC
BP
GeneRatio
Additional File 7: Figure S3. GO analysis of DEGs between the OsMBD707-overexpression line OX707-#21 and wild-type
