# Supplementary material for: Overexpression of a methyl-CpG-binding protein gene OsMBD707 leads to larger tiller angles and reduced photoperiod sensitivity in rice
Source: BMC Plant Biol. 2021 Feb 18;21:100. doi: 10.1186/s12870-021-02880-3 (PMC7893954; doi:10.1186/s12870-021-02880-3)
Supplement: Supplementary file 11 — Additional file 11: Figure S5. Expression profiles of tiller angle regulator genes in OsMBD707-overexpression plant, and of OsMBD707 under short day (SD) and long day (LD). (PPT 2596 kb) [file 12870_2021_2880_MOESM11_ESM.ppt]

## Slide 1
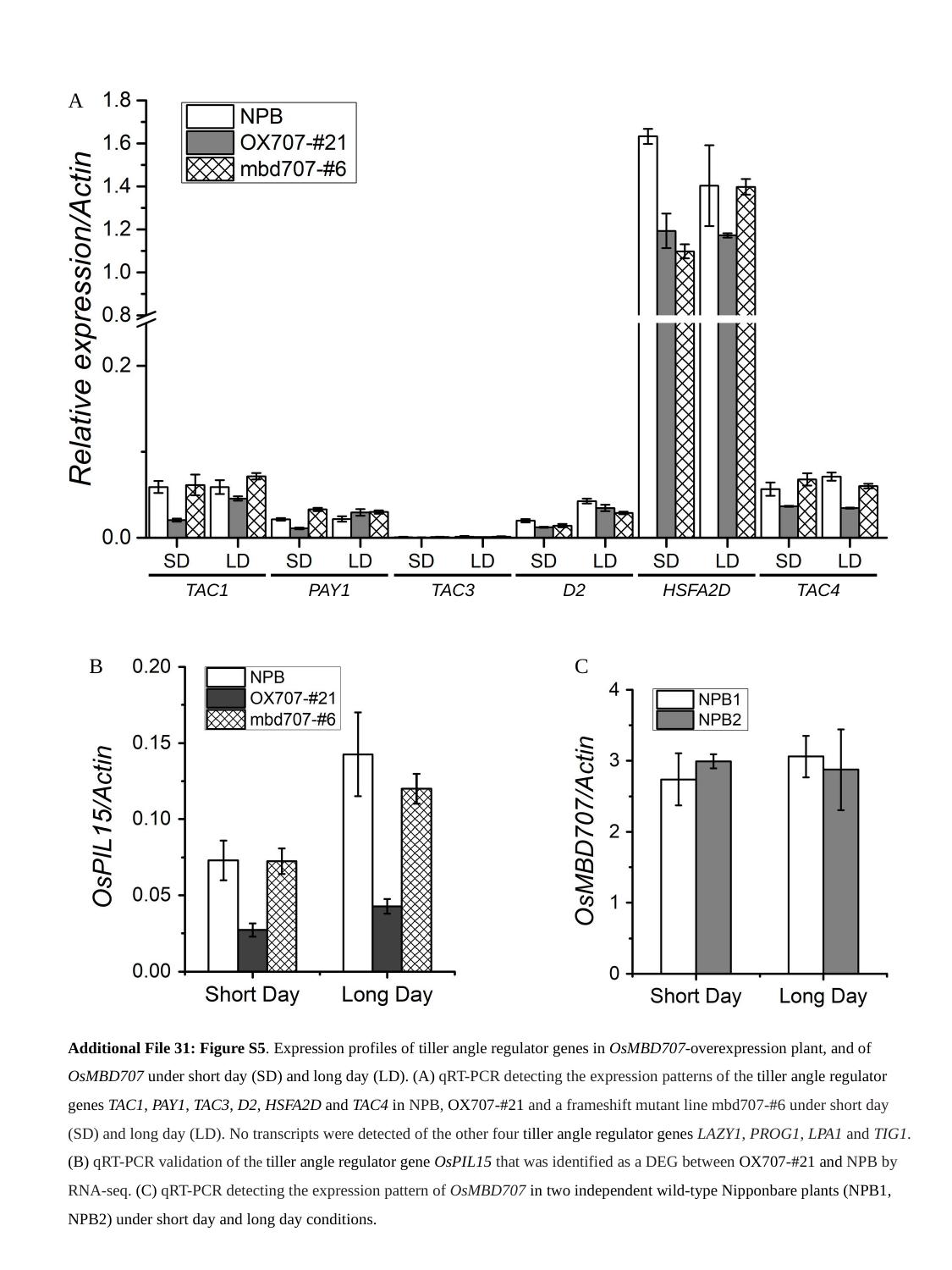

A
TAC1
PAY1
TAC3
D2
HSFA2D
TAC4
B
C
Additional File 31: Figure S5. Expression profiles of tiller angle regulator genes in OsMBD707-overexpression plant, and of OsMBD707 under short day (SD) and long day (LD). (A) qRT-PCR detecting the expression patterns of the tiller angle regulator genes TAC1, PAY1, TAC3, D2, HSFA2D and TAC4 in NPB, OX707-#21 and a frameshift mutant line mbd707-#6 under short day (SD) and long day (LD). No transcripts were detected of the other four tiller angle regulator genes LAZY1, PROG1, LPA1 and TIG1.
(B) qRT-PCR validation of the tiller angle regulator gene OsPIL15 that was identified as a DEG between OX707-#21 and NPB by RNA-seq. (C) qRT-PCR detecting the expression pattern of OsMBD707 in two independent wild-type Nipponbare plants (NPB1, NPB2) under short day and long day conditions.
