# Supplementary material for: Overexpression of a methyl-CpG-binding protein gene OsMBD707 leads to larger tiller angles and reduced photoperiod sensitivity in rice
Source: BMC Plant Biol. 2021 Feb 18;21:100. doi: 10.1186/s12870-021-02880-3 (PMC7893954; doi:10.1186/s12870-021-02880-3)
Supplement: Supplementary file 12 — Additional file 12: Table S7. Primers used in this study. [file 12870_2021_2880_MOESM12_ESM.docx]

**Additional file 12: Table S7.** Primers used in this study

| **Primer** | **Sequence (5′-3′)** | **Comment** |
| --- | --- | --- |
| Actin-F | AGTGTCTGGATTGGAGGAT | Forward primer for qRT-PCR analysis of *Actin* |
| Actin-R | TCTTGGCTTAGCATTCTTG | Reverse primer for qRT-PCR analysis of *Actin* |
| OsMBD701-F | GTTGGAGGCTGAATCTGACT | Forward primer for qRT-PCR analysis of *OsMBD701* |
| OsMBD701-R | GCTAATCTTACACGGCAGGT | Reverse primer for qRT-PCR analysis of *OsMBD701* |
| OsMBD703-F | AGTTCATTCTCGCAAGTCTCAA | Forward primer for qRT-PCR analysis of *OsMBD703* |
| OsMBD703-R | TCACCATCACTGTCATCATCAAT | Reverse primer for qRT-PCR analysis of *OsMBD703* |
| OsMBD704-F | AGATGGTGACTCCTCTGATGA | Forward primer for qRT-PCR analysis of *OsMBD704* |
| OsMBD704-R | GGACGCTTGGCAGTGTTA | Reverse primer for qRT-PCR analysis of *OsMBD704* |
| OsMBD705-F | GAAGCAGAGGAGAAGGACAAG | Forward primer for qRT-PCR analysis of *OsMBD705* |
| OsMBD705-R | GCACACGCTCATCCATCT | Reverse primer for qRT-PCR analysis of *OsMBD705* |
| OsMBD706-F | GCAGTTCAATGTTGTGAGTGT | Forward primer for qRT-PCR analysis of *OsMBD706* |
| OsMBD706-R | CGAGCAGTCAGGTCTTCTAC | Reverse primer for qRT-PCR analysis of *OsMBD706* |
| OsMBD707-F | GGCACAGCAACATACATACATAC | Forward primer for qRT-PCR analysis of *OsMBD707* |
| OsMBD707-R | ACGGACAGACGACGGTATA | Reverse primer for qRT-PCR analysis of *OsMBD707* |
| OsMBD708-F | GAATCAATACAATGGCAGCACTC | Forward primer for qRT-PCR analysis of *OsMBD708* |
| OsMBD708-R | CTTCAACAGAACGAACCAGAGA | Reverse primer for qRT-PCR analysis of *OsMBD708* |
| OsMBD709-F | CACCAGCCGATTCCTCTC | Forward primer for qRT-PCR analysis of *OsMBD709* |
| OsMBD709-R | GCCTTCACTGACCATCCTT | Reverse primer for qRT-PCR analysis of *OsMBD709* |
| OsMBD710-F | GAATGGCTATTGACCAGATTGATG | Forward primer for qRT-PCR analysis of *OsMBD710* |
| OsMBD710-R | GGCACCTAATGACCTCTTCTC | Reverse primer for qRT-PCR analysis of *OsMBD710* |
| OsMBD711-F | GGTGATGGCGAAGTCTGAG | Forward primer for qRT-PCR analysis of *OsMBD711* |
| OsMBD711-R | TTGCTGCTGGAACTTGGAA | Reverse primer for qRT-PCR analysis of *OsMBD711* |
| OsMBD714-F | GAGGTCTATCGTGGTGATAATGG | Forward primer for qRT-PCR analysis of *OsMBD714* |
| OsMBD714-R | CAGCCTACAGCACAGTTCTT | Reverse primer for qRT-PCR analysis of *OsMBD714* |
| OsMBD715-F | ATGACCACGGAGGAGGAG | Forward primer for qRT-PCR analysis of *OsMBD715* |
| OsMBD715-R | GAAGAAGGACGCATCAACTAACT | Reverse primer for qRT-PCR analysis of *OsMBD715* |
| OsMBD717-F | GGAGACGCAACGAGACAA | Forward primer for qRT-PCR analysis of *OsMBD717* |
| OsMBD717-R | GCAGCAAGAACACGGTAGA | Reverse primer for qRT-PCR analysis of *OsMBD717* |
| OsMBD718-F | CCTGAAGATGAACTTATCGGTGTA | Forward primer for qRT-PCR analysis of *OsMBD718* |
| OsMBD718-R | GGTGTCTGCTTGGCTTGA | Reverse primer for qRT-PCR analysis of *OsMBD718* |
| Os04g0192775-F | ATGGCTGGATAATGGAGGTCTAT | Forward primer for qRT-PCR analysis of putative *Os04g0192775* |
| Os04g0192775-R | GTGTCTCACCGTAAGGTTACTG | Reverse primer for qRT-PCR analysis of putative *Os04g0192775* |
| LOC_Os04g11510-F | AGTGATGATGAGAATGTGGATGAG | Forward primer for qRT-PCR analysis of putative *LOC_Os04g11510* |
| LOC_Os04g11510-R | GATAATACAACACCTCTGGCTTCA | Reverse primer for qRT-PCR analysis of putative *LOC_Os04g11510* |
| 707-1/2-F | GAAGGAGGTGGTGTCCGTG | Forward primer for detecting putative alternative transcript of *OsMBD707* |
| 707-1/2-R | TCCAGTTGGTGAAACAAAAACA | Reverse primer for detecting putative alternative transcript of *OsMBD707* |
| Ehd1-F | GGTCAACTACAATGGCGAGAT | Forward primer for qRT-PCR analysis of *Ehd1* |
| Ehd1-R | TCTTCTCCGAGGTGGTTCA | Reverse primer for qRT-PCR analysis of *Ehd1* |
| Hd3a-F | ATCACAGTATATTTGCTCCCTGC | Forward primer for qRT-PCR analysis of *Hd3a* |
| Hd3a-R | CTTCCGGCCATCGATCTTG | Reverse primer for qRT-PCR analysis of *Hd3a* |
| RFT1-F | TCGCCACCGTCTACTTCA | Forward primer for qRT-PCR analysis of *RFT1* |
| RFT1-R | TACAGCTAGGCAGGTCTCAG | Reverse primer for qRT-PCR analysis of *RFT1* |
| OsMADS14-F | CAAGTTCCTCATCATCCTCCTT | Forward primer for qRT-PCR analysis of *OsMADS14* |
| OsMADS14-R | TGCTGCTACATCCTCTATCCT | Reverse primer for qRT-PCR analysis of *OsMADS14* |
| OsMADS15-F | CCGAGAGTGAGGGAAATTGG | Forward primer for qRT-PCR analysis of *OsMADS15* |
| OsMADS15-R | GGAGTTCTTTGAGATTCAGGGATT | Reverse primer for qRT-PCR analysis of *OsMADS15* |
| OsMBD707P-F | AGGGTAGTGGTTTTCTTGTCTG | Forward primer for cloning *OsMBD707* promoter |
| OsMBD707P-R | CCATGGTTTCGAGTTTTCGCT | Reverse primer for cloning *OsMBD707* promoter |
| OX-MBD707-F | AAAACTCGAAACCATGGCCACG | Forward primer for cloning *OsMBD707* ORF |
| OX-MBD707-R | TTCAGGTGCACTTCACGGCA | Reverse primer for cloning *OsMBD707* ORF |
| MBDi-F | GGAGGTGGTGTCCGTGGAGAT | Forward primer for amplifying *OsMBD707* fragment to make RNAi construct |
| MBDi-R | GCAGAGCGCCTTGGAGTATCA | Reverse primer for amplifying *OsMBD707* fragment to make RNAi construct |
| GL-Mi-F | ACTCCAAGGCGCTCTGCATCTACCCGCTTC | Forward primer for amplifying *GUS* linker fragment to make RNAi construct |
| GL-Mi-R | ACTCCAAGGCGCTCTGCTAATCGCCTGTAAG | Reverse primer for amplifying *GUS* linker fragment to make RNAi construct |
| MBD-Cas-F1 | CAGTTTCGCTTCTTCATTTTCGG | Forward primer for sgRNA oligo1 to make CRISPR/Cas9 construct |
| MBD-Cas-R1 | AACCCGAAAATGAAGAAGCGAAA | Reverse primer for sgRNA oligo1 to make CRISPR/Cas9 construct |
| MBD-Cas-F2 | CAGTATCTGAAGGCACACCCTGG | Forward primer for sgRNA oligo2 to make CRISPR/Cas9 construct |
| MBD-Cas-R2 | AACCCAGGGTGTGCCTTCAGATA | Reverse primer for sgRNA oligo2 to make CRISPR/Cas9 construct |
| NGFP-F | AGATCTATGGTGAGCAAGGGCGA | Forward primer for *GFP* to make *pCS-NGFP* |
| NGFP-R | GGATCCCTTGTACAGCTCGT | Reverse primer for *GFP* to make *pCS-NGFP* |
| TAC1-F | AAGGTGTTCAATTGGCTGAATC | Forward primer for qRT-PCR analysis of *TAC1* |
| TAC1-R | CAGAGAGTCTTCCTTCTCTTCC | Reverse primer for qRT-PCR analysis of *TAC1* |
| D2-F | CCAACTGGAAGAGGAGAACATA | Forward primer for qRT-PCR analysis of *D2* |
| D2-R | ACATGTAGTCTGTCCATTGCAA | Reverse primer for qRT-PCR analysis of *D2* |
| OsHSFA2D-F | TGTCTTATCTCCCTGGATCTCA | Forward primer for qRT-PCR analysis of *OsHSfa2D* |
| OsHSFA2D-R | GCCTGTCGATCTCTTCATCTAA | Reverse primer for qRT-PCR analysis of *OsHSfa2D* |
| OsPIL15-F | AACACTGGAGCACTTCAGATG | Forward primer for qRT-PCR analysis of *OsPIL15* |
| OsPIL15-R | CAAAGATGCAAATGGTGGTACA | Reverse primer for qRT-PCR analysis of *OsPIL15* |
| TAC4-F | GAGAACAAGCACCATTGGATG | Forward primer for qRT-PCR analysis of *TAC4* |
| TAC4-R | CTCCTGGATTTGGTGCTTGT | Reverse primer for qRT-PCR analysis of *TAC4* |
| PAY1-F | GCTTTCGAATCAAGAAGGGTAC | Forward primer for qRT-PCR analysis of *PAY1* |
| PAY1-R | CCACATGATCCGTATACCTCAA | Reverse primer for qRT-PCR analysis of *PAY1* |
| LPA1-F | CGTTCTCGTCCAACTCCAC | Forward primer for qRT-PCR analysis of *LPA1* |
| LPA1-R | CTGCAGCTTCGTCGTTGTC | Reverse primer for qRT-PCR analysis of *LPA1* |
| T1G1-F | CTCCTTACCCTAACAACAACCT | Forward primer for qRT-PCR analysis of *T1G1* |
| T1G1-R | AAGCTGGTGGATGTAATGGTAA | Reverse primer for qRT-PCR analysis of *T1G1* |
| LAZY1-F | CACGACAACGACTACTTCTCC | Forward primer for qRT-PCR analysis of *LAZY1* |
| LAZY1-R | GAAGTCGATGTCGTAGTCGTC | Reverse primer for qRT-PCR analysis of *LAZY1* |
| PROG1-F | TCTGTAGCTAGCGTCTACTACT | Forward primer for qRT-PCR analysis of *PROG1* |
| PROG1-R | CAAACGAGAGATCGATCGAGTA | Reverse primer for qRT-PCR analysis of *PROG1* |
| TAC3-F | CGCCTTCTTCTTCTCCAGAG | Forward primer for qRT-PCR analysis of *TAC3* |
| TAC3-R | ACTTCCAGTTCAACGGCTTGTA | Reverse primer for qRT-PCR analysis of *TAC3* |
